# Uncovering Novel lncRNAs Linked to Melanoma Growth and Migration with CRISPR Inhibition Screening

**DOI:** 10.1158/2767-9764.CRC-24-0416

**Published:** 2025-07-09

**Authors:** Stavroula Petroulia, Kathryn Hockemeyer, Shashank Tiwari, Pietro Berico, Sama Shamloo, Seyedeh Elnaz Banijamali, Eleazar Vega-Saenz de Miera, Yixiao Gong, Palaniraja Thandapani, Eric Wang, Jeffrey L. Schloßhauer, Aristotelis Tsirigos, Iman Osman, Ioannis Aifantis, Jochen Imig

**Affiliations:** 1 Chemical Genomics Centre of the Max Planck Society Dortmund, Germany.; 2 Max Planck Institute of Molecular Physiology Dortmund, Germany.; 3 Department of Pathology and Laura & Isaac Perlmutter Cancer Center, NYU School of Medicine, New York, New York.; 4 Laura and Isaac Perlmutter Cancer Center, New York University, School of Medicine, New York, New York.; 5 Interdisciplinary Melanoma Cooperative Group (IMCG), NYU Perlmutter Cancer Center, NYU Langone Health, New York, New York.; 6 Department of Urology, New York University School of Medicine, New York, New York.; 7 Department of Medicine, New York University School of Medicine, New York, New York.; 8 The Ronald O. Perelman Department of Dermatology, New York University School of Medicine, New York, New York.; 9 Applied Bioinformatics Laboratories, Office of Science and Research, New York University School of Medicine, New York, New York.; 10 The Jackson Laboratory for Genomic Medicine, Farmington, Connecticut.

## Abstract

Melanoma being one of the most common and deadliest skin cancers has been increasing since the past decade. Patients at advanced stages of the disease have very poor prognoses, as opposed to at the earlier stages. Nowadays, the standard of care of advanced melanoma is resection, followed by immune checkpoint inhibition–based immunotherapy. However, a substantial proportion of patients either do not respond or develop resistance. This underscores a need for novel approaches and therapeutic targets as well as a better understanding of the mechanisms of melanoma pathogenesis. Long noncoding RNAs (lncRNA) comprise a poorly characterized class of functional players and promising targets in promoting malignancy. Certain lncRNAs have been identified to play integral roles in melanoma progression and drug resistance; however, systematic screens to uncover novel functional lncRNAs are scarce. In this study, we profile differentially expressed lncRNAs in patient-derived short-term metastatic cultures and BRAF–MEK inhibition–resistant cells. We conduct a focused growth-related CRISPR inhibition screen of overexpressed lncRNAs, validate, and functionally characterize lncRNA hits with respect to cellular growth, invasive capacities, and apoptosis *in vitro* as well as the transcriptomic impact of our lead candidate the novel lncRNA XLOC_030781. In sum, we extend the current knowledge of ncRNAs and their potential relevance in melanoma.

**Significance:**

LncRNAs have emerged as novel players in regulating many cellular aspects also in melanoma. The number of functional significances of most lncRNAs remains elusive. We provide a comprehensive strategy to identify functionally relevant lncRNAs in melanoma by combining expression profiling with CRISPR inhibition growths screens. Our results broaden the characterized lncRNAs as potential targets for future therapeutic applications.

## Introduction

Malignant melanoma of the skin represents a significant clinical challenge because of its aggressive nature and propensity for metastasis (1). Despite advances in therapeutic strategies such as targeted and immune therapies (2), melanoma remains one of the deadliest forms of skin cancer, with increasing incidence rates globally (3). The clinical management of melanoma necessitates a deeper understanding of its molecular underpinnings to improve diagnostic accuracy, prognosis, and therapeutic outcomes.

From that perspective, long noncoding RNAs (lncRNA) have emerged as pivotal regulators of gene expression and cellular processes, orchestrating diverse biological functions (4, 5). LncRNA dysregulation has been implicated in various pathologic conditions, including melanoma (6, 7), in which regulatory roles of lncRNAs are increasingly recognized, however, not fully understood. Moreover, the ability of melanoma cells to acquire invasive properties also by the involvement of lncRNAs (7–9) underscores the importance of identifying novel therapeutic targets and predictive biomarkers. LncRNAs exert regulatory effects on gene expression at multiple levels, including chromatin remodeling, transcriptional regulation, and posttranscriptional processing, thus modulating key signaling pathways implicated in melanoma pathogenesis (7). Several lncRNAs have been involved in melanoma development, progression, and therapeutic response. Notably, dysregulated expression of lncRNAs such as MALAT1, HOTAIR, and SAMMSON has been associated with melanoma metastasis, invasion, and resistance to chemotherapy (6, 10, 11) These lncRNAs function as oncogenic drivers by promoting tumor cell proliferation, migration, and epithelial–mesenchymal transition; however, our common understanding remains far from complete. Overall, elucidating the intricate interplay between melanoma and lncRNAs holds great promise for advancing our understanding of disease pathogenesis and improving clinical outcomes. Thus, improved knowledge of the regulation of melanoma pathogenesis by lncRNAs will offer novel insights into mechanisms of progression and therapeutic resistance in this disease.

In the present work, we aimed to define the extent to which lncRNAs contribute to melanoma progression by conducting an unbiased, selective, and integrative screening approach. We systematically executed a series of multistep filtering selection of lncRNAs in malignant melanoma including (i) pre-identification of overexpressed lncRNAs in clinically relevant metastatic melanoma against healthy melanocytic samples as well as multidrug-resistant versus parental cell lines, hypothesizing a potential impact in cellular fitness or survival. This could uncover eventual melanoma-specific vulnerabilities and genetic dependencies based on malignant lncRNA expression patterns, laying the groundwork of potential future druggable targets. (ii) A focused CRISPR inhibition (CRISPRi) lncRNA growth screen in melanoma cells to unambiguously identify candidates which affect cell viability. We leveraged this strategy to gain the two-fold benefit of increasing the screening hit rate while lowering the effort and screening cost as whole–lncRNA transcriptome CRISPRi analyses are challenging to conduct. Lastly, we validate and functionally characterize individual lncRNAs for growth- and invasion-related impact *in cellulo*.

## Materials and Methods

### Cell culture

Lenti-X 293T cells were purchased from Takara Bio. 501mel (RRID: CVCL_4633) and Lenti-X 293T were grown in Gibco DMEM containing high glucose with pyruvate supplemented with 10% (v/v) FBS and 1% (v/v) penicillin/streptomycin. SK-MEL239 (RRID: CVCL_6122) were kept in RPMI containing 10% FBS. SK-MEL239 2× resistant cells were kept passage-wise on-off with trametinib (1.5 nmol/L, Selleckchem) and vemurafenib/PLX4032 (2 µmol/L, Thermo Fisher Scientific) in RPMI containing 10% FBS. WM1361A (RRID: CVCL_6788) were cultured in medium containing 80% (v/v) MCDB153 with trace elements, L-glutamine and 28 mmol/L HEPES, and 20% (v/v) Leibovitz L-15 and supplemented with 1.2 g/L NaHCO_3_, 2% heat-inactivated FBS, 1.68 mmol/L CaCl_2_, 5 μg/mL insulin from bovine pancreas, and 1% (v/v) penicillin/streptomycin. All cells were kept under 5% CO_2_ and at 37°C with regular *Mycoplasma* checks (every 3–4 month using Sigma-Aldrich LookOut Mycoplasma qPCR kit, MP0040A). Short-term cultures (STC) were established at NYU Langone Medical Center (Iman Osman lab) from surgically resected patient tissues including detailed information of generation, culture conditions, karyotyping, and mutational profiles, etc. (12). Written informed consent was obtained from all participating patients according to the Declaration of Helsinki and approved by the NYU Langone internal ethical review board. Melanocytes were retrieved from commercial vendors and kept under their recommended conditions (PromoCell, see Hanniford and colleagues for details; ref. 13). All cell lines were authenticated by short tandem repeat PCR at lab entry.

### Lentivirus production and stable cell line generation

Lentiviral particles were produced by a standard transfection protocol. In a 150-mm cell culture dish, 10^7^ Lenti-X 293T cells were plated. Transfection was performed with psPAX2 (viral packaging plasmid, RRID: Addgene_12260) and pCMV-VSV-G (viral envelope plasmid, RRID: Addgene_8454) and the respective cargo delivery plasmid using polyethyleneimine transfection reagent. Virus supernatants were harvested up to 3 days after infection and stored at 4°C. Cleared supernatants were filtered through a 0.2-μm filter, concentrated using a concentrating filter, and stored at −80°C until use. Target cell lines were plated each in six-well plates using a seeding density of 2.5 × 10^5^ cells/well until ∼70% confluency. The cell growth medium was changed to fresh culture medium containing 8.0 μg/mL of polybrene and 50 to 75 μL of concentrated single-guide RNA (sgRNA) lentivirus. Puromycin was added to the cells 48 hours after infection in order to select stable modified cell lines (2.0 μg/mL). Stable dCas9 expression was detected by mCherry fluorescence flow cytometry, qPCR, and Western blotting using mouse mAb (2-2.2.14) mouse anti-HA (Thermo Fisher Scientific, #26183, RRID: AB_2533056) or mouse mAb anti-Cas9 antibody (7A9-3A3, Cell Signaling Technology, RRID: AB_2750916). Secondary antibody was anti–mouse IGG–HRP (A9044, Sigma-Aldrich, RRID: AB_258431).

### Apoptosis assay

Expression lncRNA candidates brain-derived neurotrophic factor antisense strand RNA (BDNF-AS), GDP-mannose 4,6-dehydratase antisense 1 (GMDS-AS1), and XLOC_030781 were downregulated with CRISPRi alongside sgROSA nontargeting control in 501-mel-tetON dCas9 KRAB and WM1361A-tetON dCas9 KRAB as described below using best performing sgRNAs in Supplementary Table S3. dCas9-KRAB cell lines were cultured in the presence of 2 μg/mL doxycycline (Dox) to induce the expression of dCas9, and subsequently 2.0 × 10^5^ cell/well were seeded in six-well cell culture plates. On the following day, the cells were infected with 50 μL sgRNA lentivirus of the respective lncRNAs in separate wells. Then 72 hours after infection, 150 µmol/L of etoposide apoptosis inducer was added to untreated cells as positive control. The cells were infected with sgRNA, selected with puromycin (2 μg/mL) for 48 hours, washed with PBS, and then lysed. Samples were used for immunoblotting according to (14) and probed with primary goat anti-PARP antibody (Cell Signaling Technology, #9542S, RRID: AB_2160739) and goat anti–human IgG antibody HRP conjugate (Sigma-Aldrich, #AP112P, RRID: AB_90720). Mouse monoclonal anti-GAPDH antibody (Proteintech, 1E6D9, #60004, RRID: AB_2919223) was applied as total protein loading control.

### Cell cycle distribution assay

The cells were treated as described above. The cells were washed with 1× PBS and collected in FACS tubes 72 hours after infection. Then 3 mL ice-cold 70% ethanol was added dropwise in the cell solution while mixing and incubated for 1 hour at 4°C. Then, the cells were washed twice with 1× PBS, mixed with 200 μL staining solution containing 7-Aminoactinomycin D (7-AAD) (BioGem, #61410-00) according to the manufacturer’s recommendation, and incubated for 30 minutes at room temperature protected from light. The cells were washed twice with 1 mL 1× PBS, resuspended in 300 μL flow buffer (1× PBS/FBS 2% and 2 mmol/L EDTA), and subjected flow cytometry analysis (Sony, SH800S Cell Sorter or BD FACSAria). Cell cycle analysis of vital mCherry-dCas9–expressing and GFP-sgRNA–expressing double-positive cells was done using FlowJo Software (RRID: SCR_008520).

### GFP competition assay

Assay was done in analogy to (15) using described sgRNA RPA3 positive control and best performing sgRNAs from CRISPR screen 1.0 (Supplementary Table S3). In brief, lncRNAs were knocked down in 501-mel-tetON dCas9 KRAB or WM1361A-tetON dCas9 KRAB for up to 24 days after infection and the GFP+ cell population was quantified at time points day 8, 12, 18, 20, and 24 (501mel) or day 4, 8, and 14 (WM1361a) using flow cytometry normalized to day 4 assay start control and sgROSA. The cells were gated for vital singlets expressing mCherry-dCas9.

### Transwell migration assay

The CRISPRi 501-mel-tetON dCas9 KRAB cell line was used to knock down expression of BDNF-AS, GMDS-AS1, and XLOC_030781 for 72 hours along with sgROSA control using the best sgRNA for each. FluoroBlok 24-well inserts with 8.0-µm colored PET Membrane were coated with Matrigel at a final concentration of 300 μg/mL in coating buffer solution (0.01 mol/L Tris–HCl pH 8.0 and 0.7% NaCl) for 2 hours at 37°C. In 300 μL serum-free growth medium, 1.4 × 10^5^ cells were seeded per insert and incubated for 48 hours. The lower chamber was filled with 700 μL complete growth medium with 1 µmol/L lysophosphatidic acid chemoattractant. Invading live cells were stained with 500 μL per insert calcein AM 2 μg/mL in Hank’s Balanced Salt Solution 1× for 10 minutes at 37°C before inverted fluorescent microscopy imaging was performed. ImageJ (Fiji, RRID: SCR_003070) software was employed to quantify migrating cells. The SAM-dCas9 501Mel cell line was generated following the previously described method (16) by seeding 5 × 10^5^ cells per well instead.

### qPCR

qPCR was done according to (17). Typically, 1 µg of total RNA was reverse-transcribed with the High-Capacity cDNA Reverse Transcription Kit (Thermo Fisher Scientific, #4368814), and qPCR was performed with GoTaq (Promega, #M3001) according to the manufacturer’s instructions with primer sets listed in Supplementary Table S5 and analyzed likewise using the 2^–∆∆*Ct*^ method (18).

### Comet assay

Cell lines used for the assay were 501-mel-tetON dCas9 KRAB or WM1361A-tetON knocked down with sgXLOC_030781_6 for 3 and 5 days after puromycin selection start. The Alkaline CometAssay Kit from R&D Systems (#4250-050-K) was used according to the manufacturer’s instructions. H_2_O_2_-treated cells were used as positive and untreated cells as negative controls. Fluorescently stained cell tails were microscopically quantified using Fiji software in percent to positive control.

### sgRNA library construction

sgRNAs were placed in a window −300 to +50 bp up- and downstream of identified transcription start site applying http://benchling.com (RRID: SCR_013955) with off-target score of 70 or higher. Library-targeted 262 lncRNAs were upregulated in metastatic STCs versus melanocytes and BRAF inhibition (BRAFi) and MEK inhibition (MEKi)–resistant SK-MEL239 (RRID: CVCL_6122) cells, including in total 2,761 sgRNAs (10 sgRNAs per lncRNA gene), 50 scrambled sgRNA controls, and five lncRNA positive controls (ANRIL, BANCR, HOTAIR, MALAT1, and SAMMSON). Individual sgRNA oligos were synthesized by Twist Bioscience (https://twistbioscience.com/) on a 12K array and amplified using array primers (Supplementary Table S5). Using a Gibson Assembly master mix (New England Biolabs), we cloned sgRNAs into a lentiviral sgRNA GFP-tagged vector (LRG; Addgene, #65656). Gibson reactions were transformed using DH10B electrocompetent cells (Invitrogen) at 2 kV, 200 Ω, and 25 µF. Bacterial colonies were quantified to obtain ∼70× coverage. Subsequently, the library was deep-sequenced using MiSeq to confirm sgRNA representation. All sgRNA sequences used in this study are provided in SupplementaryTable S2 including extended information of important and lncRNA-positive controls related to melanoma background. CRISPRa sgRNA design was done in accordance with (16).

### CRISPRi screen and data analysis

In brief, 501mel and WM1361a cells were transduced with an in-house–generated Gibson-assembled vector containing KRAB-dCas9-HA-Tag and mCherry fluorescence marker separated by a P2A cleavage signal under the Dox-inducible TRE3G promoter in a lentiviral packing signal backbone. Additionally, we cloned in an opposite direction upstream of the TRE3G blasticidin antibiotic marker and rtTA tetracycline resistance gene under control of the constitutive PGK promoter. Lentivirus-transduced, stably selected, and mCherry-positive 501mel cells were used for screening (single clones D8 and D12 replicates for screen 1.0 and two cell pool replicates for screen 2.0). dCas9 expression check of 501mel was confirmed as in Supplementary Fig. S2. Then ∼9.2 × 10^6^ cells were infected with the LRG-GFP sgRNA library expression vector at a multiplicity of infection 0.1 to 0.3 to achieve 1,000× coverage. After 1 day of recovery, infected cells were puromycin (2 µg/mL) selected for 3 days under simultaneous Dox induction (screen 1.0) or Dox was added (each 2 µg/mL) after puromycin end selection after 3 days. Then puromycin was removed, followed by Dox addition on day 4 in order to prevent premature drop-off (screen 2.0). Dox induction lasted for at least 2 days until screening start time point. One day before screening start, the cells were sorted near 100% positivity for mCherry and GFP double-positive cells maintaining 1,000× coverage before each screen as in 2.2 to 2.3. At time points day 7, 14, and 21 after screen start, 1,000× cells of library size were collected and genomic DNA (gDNA) was extracted with the QIAamp DNA Blood Mini Kit (Qiagen, #511049) and cleaned up with OneStep PCR Inhibitor Removal Kits (Zymo, #D6030) to remove melanin as a potent PCR inhibitor. Additionally, we ran in parallel non–Dox-treated cells as negative control to rule out stochastic sgRNA loss or overrepresentation. Keeping an equivalent of 1,000× coverage gDNA quantity for each sample, the integrated sgRNA library was PCR amplified with limiting cycles using barcoded primers listed in Supplementary Table S5. Subsequently, amplified fragments were sequenced using Illumina HiSeq 2000 to yield roughly 1,000 reads per guide RNA. Analysis was done similar to (19) by counts per million (CPM) counting sgRNA reads of all screening time points (screen 1.0) or log_2_ fold change (fc) read counts (screen 2.0) against control day 4 (screening start). Hit selection from screen 1.0 was done by calculating the median log_2_ fc of top three depleted sgRNAs per lncRNA at day 21 (i.e., end time point).

### RNA sequencing and analysis

RNA sequencing (RNA-seq) libraries were generated using the QIAseq Stranded Total RNA Library Kit including the QIAseq FastSelect rRNA HMR Kit and QIAseq UDI Y-Adapter Kit (Qiagen, #180743, #334385, and #180310) according to the provider’s protocol. qPCR confirmed appropriate XLOC_030781 knockdown. Sequencing of the RNA libraries was performed on Illumina NovaSeq 6000 PE150 with at least 30 million reads output per sample. R version 4.3.1 was used for all analyses. Raw fastq files were processed using the Zarp pipeline (https://github.com/zavolanlab/zarp; ref. 20), which uses FastQC, zpca, and MultiQC (FastQC, RRID: SCR_014583; ref. 21) for quality control, and the adapters were trimmed using Cutadapt (22). The reads were mapped to the human genome (hg38, Genome Reference Consortium GRCh38) using STAR (RRID: SCR_00446; ref. 23) and quantized Salmon (24). The final output is a counts matrix which is used as input for the R package DESeq2 (RRID: SCR_000154; ref. 25) to identify differentially expressed genes. Genes with a log fc >1 and adjusted *P* value < 0.05 were used for further analysis. The volcano plot was generated using the EnhancedVolcano R package (26), and the ComplexHeatmap (27) package was used for generating the heatmaps. The gene sets were obtained using the msigdbr (28) R package, and clusterProfiler (RRID: SCR_016884; ref. 29) and enrichplot (30) were used for generating the gene ontology (GO) and gene set enrichment plots.

### Chromatin immunoprecipitation sequencing and analysis

Chromatin immunoprecipitation sequencing (ChIP-seq) was done in duplicates according to (31) with modifications. In brief, 1 × 10^6^ nuclei were fixed in 1% formaldehyde in PBS and incubated at room temperature for 10 minutes and thereafter quenched with 0.125 mol/L glycine final concentration. Cells were lysed with buffer containing 5 mmol/L HEPES, pH 8.0, 85 mmol/L KCl, and 0.5% IGEPAL supplemented with protease inhibitors (Roche), incubated on ice for 10 minutes, and spun to pellet the nuclei. To generate the mononucleosomal particles, the pellet was resuspended in MNase digest buffer (10 mmol/L NaCl, 10 mmol/L Tris, pH 7.5, 3 mmol/L MgCl_2_, and 1 mmol/L CaCl_2_) with 1 U of micrococcal nuclease (USB) and incubated at 37°C for 45 minutes. The reaction was stopped by adding 20 mmol/L EDTA and incubated on ice for 10 minutes. The nucleus was spun down and resuspended in nucleus lysis buffer (50 mmol/L Tris–HCl, pH 8.1, 10 mmol/L EDTA, pH 8.0, and 1% SDS) supplemented with proteinase inhibitors (Roche). Then, the samples were sonicated using a Bioruptor (Diagenode) at high intensity for five cycles (30 minutes on/30 minutes off) at 4°C to obtain sheared chromatin fragments. Magnetic Protein G beads (Dynabeads) were prepared by washing them with citrate–phosphate buffer (25 mmol/L citric acid and 66 mmol/L Na_2_HPO_4_, pH 5.0) and blocking them with 10 mg/mL BSA in citrate–phosphate buffer for 1 hour. A total of 25 µL of beads per sample and 20 µL for pre-cleaning were prepared using this method. Approximately 200 µg of chromatin fragments were pre-cleaned with 20 µL of blocked beads and 10 volumes of immunoprecipitation (IP) dilution buffer (167 mmol/L NaCl, 1.1% Triton X-100, 0.01% SDS, 1.2 mmol/L EDTA, pH 8.0, and 16.7 mmol/L Tris–HCl, pH 8.0) for 1 hour at 4°C. From the chromatin, 1% of the input was reserved. Subsequently, 25 µL of blocked beads were coupled with 5 µg of Acetyl-Histone H3 (Lys27; D5E4) XP Rabbit mAb or Tri-Methyl-Histone H3 (Lys4; C42D8) Rabbit mAb (Cell Signaling Technology) or Mono-Methyl-Histone H3 (Lys4; D1A9) Rabbit mAb (Cell Signaling Technology) for 4 hours at 4°C in IP dilution buffer. The antibody-coupled beads were added to the pre-cleaned chromatin in IP dilution buffer and incubated overnight at 4°C. After IP, DNA elution was performed by on-bead Proteinase K digest (Ambion) and overnight incubation at 65°C under high agitation. Eluted DNA was then precipitated using ethanol and glycogen. cDNA libraries were generated as described using the Kapa HyperPrep Kit (Roche and Illumina TruSeq system) and sequenced with NextSeq 500. ChIP-seq datasets were analyzed with the HiC-bench platform (32). The ChIP-seq–aligned reads were further filtered by discarding reads with low mapping quality (MAPQ <20) and duplicated reads using Picard Tools (https://github.com/broadinstitute/picard and https://github.com/NYU-BFX). The remaining reads were analyzed by applying the peak-calling algorithm MACS2 (version 2.0.1) 16 with input as control. Binding of histone marks was determined from broad peak calls.

### Transcription factor enrichment analysis

Significantly over- and underexpressed genes (log_2_ fc < 1 and >1; adjusted *P* value = 0.05) from RNA-seq of sgXLOC_030781 knockdown at day 3 were subjected to ENCODE and ChEA (RRID: SCR_005403) consensus transcription factor (TF) analysis from ChIP-X (Keenan and colleagues; ref. 33). The TF enrichment output was plotted as −log_10_ (*P* value).

### ENSG00000287723 expression analysis from available datasets

Pan-cancer expression analysis (*n* = 5,606 across 23 cancer entities) of nevi and primary melanoma for ENSG00000287723 was done by retrieving fastq files from Badal and colleagues, (GSE98394; ref. 34), Kunz and colleagues, (GSE112509; ref. 35), and The Cancer Genome Atlas (TCGA; GDC Data Portal access granted from Dr. David Fenyo, NYU). Fastq files were trimmed using trim galore (version 0.6.6), followed by quality control analysis with FastQC (version 0.12.1). Trimmed fastq were aligned to the GRCh38 human genome and gene transcripts were annotated with GENCODE V44 using STAR (version 2.7.7a, RRID: SCR_014966). Normalized read counts CPM or fragments per kilobase of transcripts per million were used to plot ENSG00000287723 expression for nevi against primary melanoma (Badal and Kunz) and primary melanoma against melanoma metastatic samples (TCGA). Statistical analysis employed a two-tailed unpaired t-test in GraphPad Prism.

### Software and statistics

Statistical analysis of qRT-PCR, Western blot quantification, cell invasion, luciferase and Comet assays, and finally gene expression data was performed using a two-tailed, paired Student *t* test to determine statistical significance: *, *P* < 0.05; **, *P* < 0.01; ***, *P* < 0.001 (GraphPad *t* test calculator). Adobe Illustrator and GraphPadPrism 8.0 (RRID: SCR_002798) were used. Fiji (RRID: SCR_002285) was utilized for microscopic object quantification related to call invasion and Comet assays. Flow cytometry analysis was done with FlowJo V8.7 TreeStar (BD Biosciences; https://flowjo.com; RRID: SCR_008520).

### Data availability and resources

#### RNA-seq

The RNA-seq data of melanoma STCs and cultured melanocytes can be found in the Gene Expression Omnibus (GEO) at GSE138711 (13) and GSE94488 (36).

The RNA-seq data of melanoma cell lines WM1361a (WM1361a-pWPI) and SK-MEL239 are deposited in GEO at GSE138711 (13) and 501mel (501mel-JQ1R-24hr and 501mel-JQ1R-6hr) at GSE94488, (36). The RNA-seq data of SK-MEL239_2x (BRAFi and MEKi resistant) of this publication are deposited at https://edmond.mpg.de/dataverse/edmond under https://doi.org/10.17617/3.5S92GO and 501mel XLOC303781_KD_RNA-seq data under https://doi.org/10.17617/3.5S92GO.

#### ChIP-seq histone modifications

The ChIP-seq data of melanoma STCs can be found in GEO at GSE60666 (37). The ChIP-seq data of cultured melanocytes can be found in GEO at GSE94488 (36). The ChIP-seq data of the melanoma cell line 501mel are in GEO at GSE94488 (36). The ChIP-seq data of melanoma cell lines WM1361a, SK-MEL239, and SK-MEL239_2x of this publication are deposited at https://edmond.mpg.de/dataverse/edmond under https://doi.org/10.17617/3.5S92GO.

#### CRISPR library screen sequencing

The CRISPRi screen 1.0 and 2.0 data of this publication can be found under https://doi.org/10.17617/3.5S92GO at https://edmond.mpg.de/dataverse/edmond, which is a large public data repository provided by the Max Planck Society.

## Results

### LncRNA candidate selection for CRISPRi screen and expression of lncRNAs associated to melanomagenesis

Whole-transcriptome functional CRISPR screening for lncRNAs is elaborate, requiring large resources given the number of annotated lncRNAs reaching more than tens of thousands (38, 39). Additionally, lncRNA functions are strictly context dependent and thus not all known lncRNAs are relevant in a model system of interest (39, 40). To overcome these challenges, it seems plausible to preselect melanoma-specific lncRNAs for a functional CRISPRi-focused screening based on differential gene expression (DGE). We sequenced five samples of lymph node (LN) and five brain metastasis patient-derived STCs against healthy donor–derived cultured melanocytes from NYU Langone Medical Center, Department of Pathology, within our Melanoma Program. For convenient consecutive melanoma cell line–based CRISPRi screen, we added differential RNA-seq expression analysis of the 501mel metastatic cell line (*BRAF*^V600E^) and WM1361a cell line with metastatic capacity (*NRAS*^Q61R^) to ensure ample expression of STC-derived DGE lncRNAs in a stable model background. Additionally, we profiled the differential expression of lncRNAs within another clinically relevant setting namely dual BRAFi and MEKi resistance phenotype in SK-MEL239 parental and SK-MEL239 double-resistant cell lines (SK-MEL239_2x was a kind gift from Dr. Poulikakos. Icahn School of Medicine, Mount Sinai). Overexpressed lncRNAs in SK-MEL239_2x were added to the CRISPRi library screen, reasoning that genes involved in targeted therapy resistance inherit roles in melanoma cell survival and can be identified by growth (16, 41).

**Figure 1:**
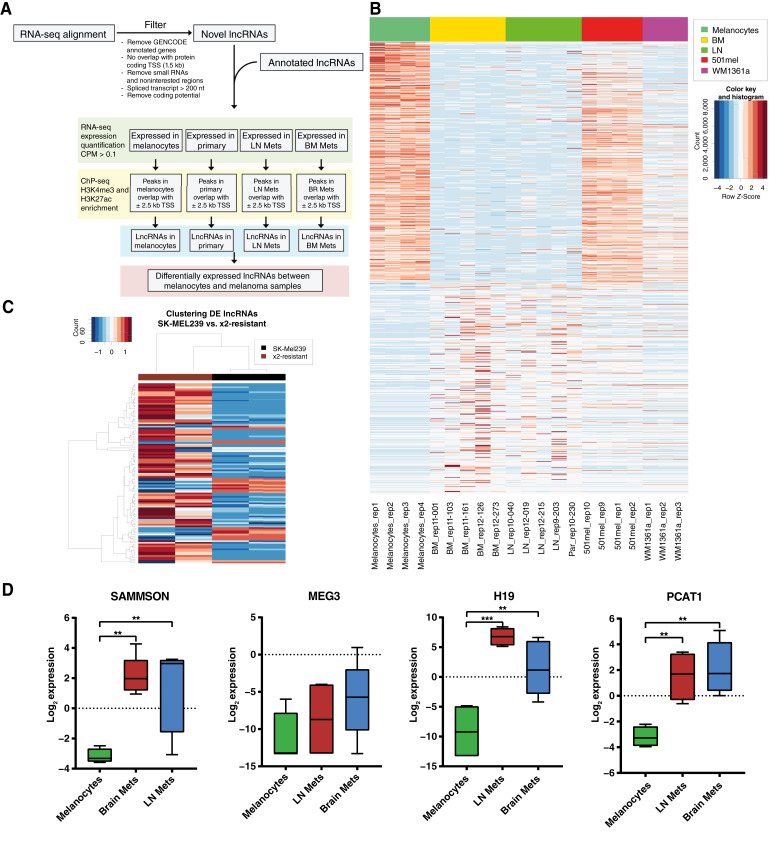
LncRNA candidate selection for CRISPRi screen and expression of lncRNAs associated to melanomagenesis. **A,** Flow diagram of integrated RNA- and ChIP-seq for the preselection of lncRNAs. Selection parameters are FDR < 5%, log fc > 0.75, and CPM > 1 in higher expressed cell type, either histone mark (H3K4me3 or H3K27ac) in the higher expressed cell type; no mono-exonic lncRNAs and log fc ≥ 0.5 between investigated cell type and either WM1361a or 501mel. **B,** Heatmap of differentially expressed lncRNAs by RNA-seq in patient/donor-derived melanocytes (*n* = 4) and metastatic STCs (BM, brain metastasis, *n* = 5; LN, lymph node metastasis, *n* = 4) and melanoma cell lines 501mel (*n* = 4) and WM1361a (*n* = 3). **C,** Heatmap showing differential lncRNA expression in the SK-Mel239 parental melanoma cell line versus BRAF and 1059 MEKi-resistant cell line (*n* = 2). **D,** Boxplot showing the log_2_ CPM expression of selected melanoma-associated lncRNAs SAMMSON, MEG3, H19, and PCAT1 in melanocytes (*n* = 4), brain metastasis (*n* = 5), and LN STCs (*n* = 4) detected by RNA-seq. DE, differentially expressed; Mets, metastasis; TSS, transcription start site.

To ensure the relevancy of the selected lncRNAs within the experimental context, specific filters and thresholds were implemented. These analytic filters encompassed (i) maintaining an FDR of less than or equal to 0.05 (FDR ≤ +0.05), (ii) adhering to a log fc threshold of 0.75 or higher (logFC > +0.75) to ascertain that the lncRNAs were significantly overexpressed across clinical samples, (iii) requiring a read count per million of at least 1 (CPM ≥ +1) in the higher expressed cellular type, excluding genes with low expression levels which are difficult to modulate by CRISPRi, (iv) mandating that the lncRNAs carried an active histone mark, specifically H3K4me3 or H3K27ac being present in the cell type in which the respective lncRNA is highly expressed compared with all others (a genome browser example of the MITF-SAMMSON lncRNA locus for melanocytes, LN, brain metastasis, and WM1361a for H3K4me and H3K27ac is given in Supplementary Fig. S1), and (v) excluding any lncRNAs that consisted solely of mono-exonic transcripts as they might execute alternate function, for example enhancer-associated RNAs (Fig. 1A). By this, we identified 88 lncRNAs to be overexpressed in brain metastasis and LN STCs versus melanocytes and 145 downregulated (Fig. 1B). In brain metastasis versus LN, only few lncRNAs seemed to be differentially expressed (brain metastasis vs. LN five lncRNAs up- and 12 downregulated), pointing toward similar lncRNA regulatory programs (Supplementary Table S1). In the comparison of melanoma cell lines SK-Mel239 versus BRAFi/MEKi-resistant derivative, we detected 174 up- and 57 downregulated lncRNAs (Fig. 1C).

We further profiled our expression data for previously described lncRNAs relevant in melanoma to assure the comprehensiveness for the following CRISPRi screen. We found that except for MEG3, all analyzed lncRNAs such as SAMMSON, H19, and PCAT1 are at least log fc >2 overexpressed in both LN and brain metastasis STCs compared with melanocytes (Fig. 1D). Overall, this indicates that the selected clinical samples exhibit a valid test cohort for pre-identification of additional potentially essential lncRNAs in melanoma pathogenesis.

### CRISPRi screen for melanoma-related lncRNAs

To functionally address the overexpressed lncRNAs found in melanoma STCs, we applied the CRISPRi system from (42, 43) in a proliferation drop-off screen. We established and implemented this system in two melanoma cell line models 501mel and WM1361a derived from metastatic lesion and primary melanoma, respectively (13, 44). Then we compiled a series of validation steps to assure full gene-repressive performance and dCas9 expression stability. After lentiviral transduction with an adapted tetON-dCas9-KRAB-mCherry vector, selection, and stable single cell line generation, we tested for robust HA-tagged dCas9-KRAB protein expression in clones derived from both cell lines by Western blotting (Supplementary Fig. S2A and S2D). Strongest expression signal could be detected in subclones D8 and D12 in 501mel. In WM1361a, two clones (F2 and F11) with sufficient dCas9-KRAB expression were generated to be used for post-screening validations in a cell line complementary to 501mel. In line with this, strong fluorescence signal of the dCas9-KRAB co-expressed mCherry marker could be detected by flow cytometry for all clones in both cell lines (Supplementary Fig. S2B and S2E). This expression signal was stable over time, enabling good CRISPRi screening conditions. CRISPRi functionality in melanoma cell lines was further confirmed by an sgRNA co-expressed with GFP in a competition assay in which two (501mel) or one (WM1361a) sgRNA was targeting the essential cell survival gene *RPA3* (15). Loss of cellular fitness of successfully infected cells was monitored over 24 days (501mel) and 14 days (WM13161a) and compared with uninfected cell population and sgROSA-negative control. The results show almost complete loss of GFP for 501mel cells whereas in WM1361a, up to ∼85% loss of the sgRNA-GFP–expressing population was seen for all selected single-cell clones (Supplementary Fig. S2C and S2F). For screen biological replicate 2.0, we employed a 501mel cell pool in two independent technical replicates and cross-confirmed transduced dCas9-KRAB–expressing cells by Western blotting (left), qPCR for Cas9 (middle), and mCherry fluorescence microscopy (right) of re-sorted cells (Supplementary Fig. S2G). Altogether, this indicates a fully functionally established CRISPRi system in melanoma cell lines being utilized for lncRNA loss-of-function cell viability screen.

Based on these findings, we built a customs library of 2,761 sgRNAs (10 per gene) against 88 lncRNAs upregulated in both brain metastasis and LN versus melanocytes and 174 lncRNAs overexpressed in BRAFi/MEKi-resistant SK-MEL239 versus parental cells. Fifty nontargeting sgRNAs were incorporated as negative controls as well as positive controls against five known melanoma-associated lncRNAs (BANCR, CDKN2B-AS1, HOTAIR, MALAT1, and SAMMSON; Supplementary Table S2). Pooled sgRNAs were subcloned into the LRG vector (45) and next-generation sequencing was carried out to confirm optimal sgRNA representation (Supplementary Fig. S3A).

**Figure 2:**
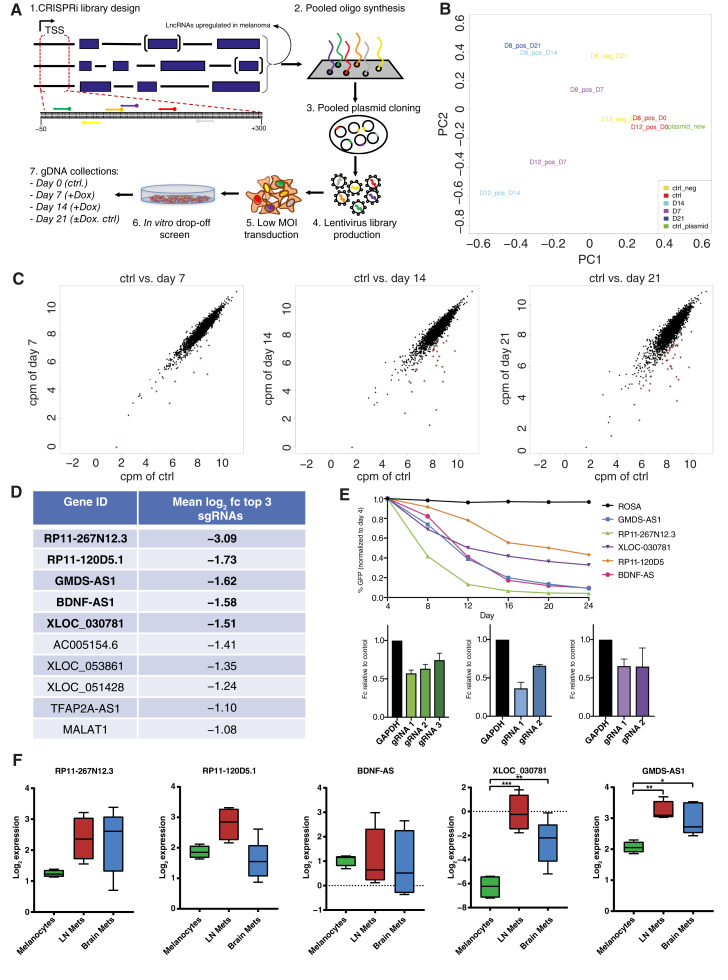
CRISPRi screen for overexpressed lncRNAs in melanoma pathogenesis and expression of selected hits. **A,** Graphical outline of the conducted CRISPRi screen in the 501mel cell line. MOI, multiplicity of infection. **B,** Principal component analysis plot of samples used for the CRISPRi screen. D8 and D12 refer to selected 501mel-dCas9-KRAB single-cell clone and D7, D14. and D21 for the days of screening time. Negative control refers to no Dox treatment, and the plasmid library for lentivirus production was added as additional control. PC, principal component. **C,** Dot plots show results of the CRISPRi lncRNA library screen at time points day 7, 14, and 21 after selection and mCherry^+^/GFP^+^ cells sort as CPM of control (= day 0) vs. respective time points. Each dot represents a single sgRNA. Red dots indicate significantly depleted sgRNAs and log_2_ fc > −1. **D,** List showing top 10 depleted lncRNA (excluding PVT1 as not validated after screen) hits at day 21 vs. control day 0 as the mean log_2_ fc of the top three depleted sgRNAs of each respective lncRNA. **E,** CRISPRi hit validation of top 2 to 3 individual sgRNAs of indicated lncRNAs; top: GFP competition assay in 501mel-dCas9-KRAB from days 4 to 24 by flow cytometry in percent GFP-positive cells. sgROSA (black curve) serves as a negative control. Curves show average GFP signal loss of sgRNAs shown in bottom: bar graphs of fold change GFP expression of individual sgRNAs relative to control at day 24. For color codes, see top panel. **F,** Boxplot showing the log_2_ CPM expression of selected lncRNA CRISPRi screening hits RP11-267N12.3, RP11-120D5.1, BDNF-AS, XLOC_030781, and GMDS-AS1 in melanocytes (*n* = 4), brain metastasis (*n* = 5), and LN STCs (*n* = 4) detected by RNA-seq. Ctrl, control; Mets, metastasis.

Subsequently, in a 501Mel dCas9-KRAB–expressing cell line, we performed a loss-of-function pooled CRISPRi screen of two consecutive independent biological replicates (referred to 1.0 and 2.0). Although in screen 1.0, replicate was comprised of 501mel cell clones D8 and D12 (Supplementary Fig. S2C), screen 2.0 consisted of a cell pool with two technical replicates (Supplementary Fig. S2G). Schemes outlining the treatment regimen and timeline are shown in Fig. 2A (screen 1.0) and Supplementary Fig. S3E (screen 2.0). After puromycin selection, PCR-amplified gDNA of Dox-induced sgRNA library was collected at days 7, 14, and 21 after screen start and subjected to deep sequencing. The end time point day 21 after screen start without Dox was analyzed as additional control to rule out stochastic library distribution changes. Thereafter, changes in sgRNA abundance were assessed by counting sgRNA reads. At first, we plotted the log_2_ CPM of all time points and technical replicates of screen 1.0 against the cloned sgRNA plasmid library, day 0 control, and no Dox control, demonstrating no major sgRNA loss or random overrepresentation (Supplementary Fig. S3B). We followed up by a hierarchical clustering analysis of all *Z*-score–normalized sgRNA counts of the same samples (Supplementary Fig. S3C). As expected, we found similar sample clustering of day 0 and no Dox or plasmid controls, whereas late time points of the screen clustered together, indicating a specific response to our negative selection CRISPRi screen perturbating the expression of the selected lncRNAs. Good replicate accordance between negative control and different time point samples was achieved with the R coefficient ranging between 0.873 and 0.959 (Supplementary Fig. S3D). Principal component analysis of the screen highlighted the similarity of all control samples on principal component 1 (PC1; ∼0.1–0.5), distinguishing them from Dox-induced samples over time points day 7 to 21 (PC1, 0–0.6), Fig. 2B. These quality checks prompted us to select for specific lncRNA hits consistently depleted over time showcasing a potential involvement in melanoma cell survival. We achieved this by plotting the CPM sgRNA read counts of day 0 negative control over all three time points of day 7, 14, and 21 of Dox-induced sgRNA expression (Fig. 2C) We implemented hit selection filter of mean log_2_ fc > −1 of the top three depleted sgRNAs per lncRNA gene at day 21 as the time point of strongest depletion. This resulted in a shortlist of 10 lncRNAs (Supplementary Fig. S2D; Supplementary Table S3), including seven previously annotated lncRNAs and three potentially novel lncRNAs (BDNF-AS, XLOC_053861, and XLOC_051428).

We validated the screen by cloning the two to three best screen performing single sgRNAs of the top five listed lncRNAs into the LRG expression vector. We then applied a competition assay in which GFP is co-expressed to the sgRNA, leading to a loss of cellular fitness in case an lncRNA inhibits cell growth. We evaluated the relative GFP-expressing 501mel cell population of five lncRNAs RP11-267N12.3, RP11-120D5, GMDS-AS1, BDNF-AS, and XLOC_030781 over 20 days in comparison with sgROSA negative control (Fig. 2E, (top) average depletion of used sgRNAs and (bottom) example selection of 2–3 sgRNAs CRISPRi efficiency targeting lncRNA expression relative to GAPDH). Our results confirmed that all sgRNAs were robustly depleted in cells over time, corroborating our screening data. We eliminated RP11-267N12.3 from our shortlist as it shares a bidirectional promoter with NUF2 mitosis–related gene (46), making this lncRNA difficult to study and distinguish its function by CRISPRi in relation to the NUF2 neighbor gene. Additionally, the previously reported oncogenic lncRNA in melanoma PVT1 was eliminated from this short list as no validation could be achieved.

To further strengthen the significance of our hit selection, we analyzed our small-panel RNA-seq expression dataset of melanocytes in LN and brain metastasis STCs (Fig. 2F, *n* = 5 and *n* = 5). We gave priority to overexpressed lncRNAs in both brain metastasis and LN metastasis lesion sites as a selection criterion, owing to a potential involvement in the process of patient’s pathophysiologic progression. Only XLOC_030781 (LN vs. melanocytes, FDR = 0.000759; brain metastasis vs. melanocytes, FDR = 0.00633; and STC vs. melanocytes, FDR = 0.00139; Supplementary Table S1) and GDMS-AS1 (LN vs. melanocytes, FDR = 0.020 and brain metastasis vs. melanocytes, FDR = 0.039; Supplementary Table S1) were significantly overexpressed in both brain metastasis and LN. However, RP11-120D5.1 showed only upregulation in LN. The RP11-267N12 and BDNF-AS transcript in trend was also elevated in LN and brain metastasis groups although not significantly overexpressed. RP11-120D5, BDNF-AS, and GMDS-AS1 were additionally found overexpressed in SK-MEL239_2x but not followed up in drug-resistant context.

To further substantiate our results, we conducted a second replicate screen 2.0 with slight modifications. This time Dox induction was carried out later at 4 days after infection and 2 days before GFP+ and mCherry+ FACS sorting in order to minimize drop-off of early sgRNA responders and, therefore, information loss (Supplementary Fig. S3E). Thereafter, the library processing and time points of sample acquisition were done in analogy to the first screen. The average sgRNA reads were calculated for each time point to the control of day 0, and the log_2_ fc of the top three sgRNAs targeting each lncRNA was plotted (Supplementary Fig. S3F). Negative control sgRNAs exhibited no significant alterations in all samples (green dots), whereas all of our previously identified lncRNA hits were re-confirmed to be depleted at all time points. Top 50 depleted lncRNAs are highlighted in purple. Strong screening reproducibility between screen 1.0 and 2.0 was achieved as shown by the high overlap of each top 50 negatively selected lncRNA hit ranks (day 7 *n* = 11, day 14 *n* = 6, and day 21 *n* = 9; Supplementary Fig. S3G). The most promising lncRNA candidates (BDNF-AS, GDMS-AS1, and XLOC_030781) that were selected from screen 1.0 maintained comparable rankings in screen 2.0, thereby reinforcing their selection in terms of robustness and significance for melanoma cell survival (Supplementary Table S3) and pointing to no prior drop-off due to Dox induction of dCas9-KRAB. Unfortunately, loss of lncRNA positive controls could only be observed in screen 2.0 for lncRNA ANRIL, potentially reflecting the strong cell type dependency of most lncRNAs (39, 40).

In conclusion, based on our screening, hit validation, novelty of lncRNA association with melanoma, and expression analyses, we carried over lncRNAs BDNF-AS, GMDS-AS1, and also XLOC_030781 to deeper phenotypic and functional characterization.

### Phenotypic and functional characterization of lncRNA candidates

Next, the major objectives of our work were to put our selected lncRNA screening hits into relation to their drop-off behavior during the CRISPRi screen by phenotypic and functional characterization in melanoma. Assuming an association of the overexpression in STCs of our lncRNA candidates with some growth-related and metastatic characteristics, we performed a series of *in vitro* cell-based assays to monitor cell-cycle progression, apoptosis induction, and cell migration in two melanoma cell lines to gain deeper insights into their potential mechanistic roles. By this, we aimed to couple both growth-related features of lncRNAs from the CRISPRi cellular fitness screen harboring in parallel to invasive capacities potentially leveraging the identification of future drug targets in melanoma.

**Figure 3:**
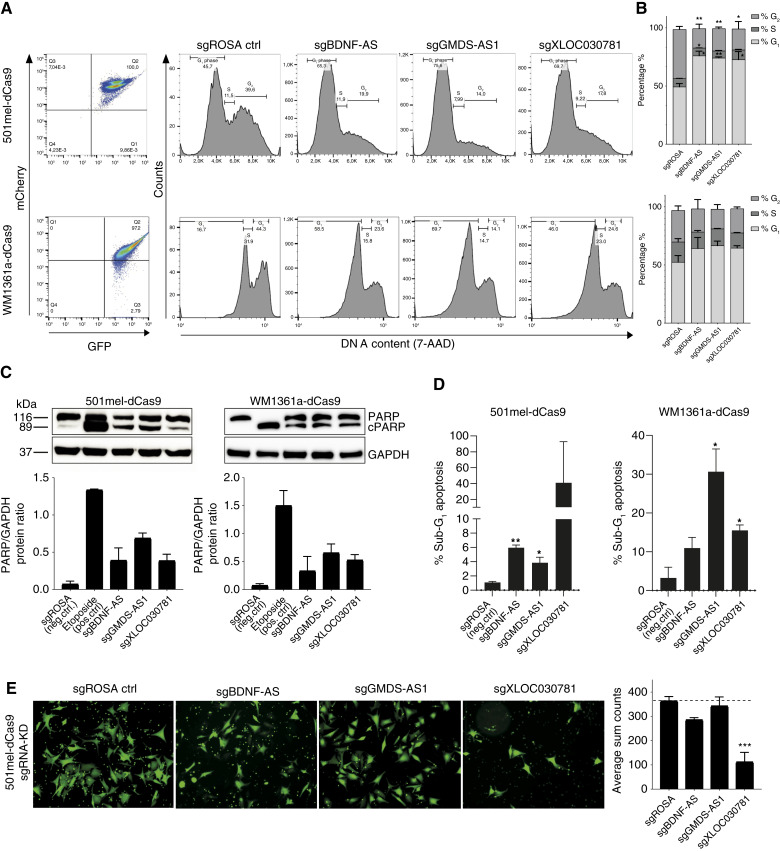
CRISPRi screen lncRNA hit functional characterization. **A,** Flow cytometry–based dotmplot showing GFP and mCherry signal as proxy for sgRNA and dCas9 expression (left). 7-AAD fluorescence–dependent cell cycle analysis in 501mel-dCas9-KRAB (top) and WM136a-dCas9-KRAB (bottom) cell lines upon sgBDNF-AS, sgXLOC_030781, and sgGMDS-AS1 CRISPRi knockdown (rigth). Note that the top panel is plotted linear and bottom logarithmic because of better cell cycle–gating parameters. **B,** Cell cycle phase quantification based on A in percent of cells (*n* = 2). **C,** PARP cleavage apoptosis assay performed by Western blotting upon sgBDNF-AS, sgXLOC_030781, and sgGMDS-AS1 knockdown in 501meld-Cas9-KRAB and WM1361a-dCas9-KRAB Representative Western blots (top). GAPDH detection was used as a loading control; densitometric quantification of apoptosis as cleaved PARP/GAPDH protein ratio (bottom; average of *n* = 2). sgROSA served as negative control and etoposide apoptosis inducer as positive control. **D,** Re-analysis of 7-AAD cell cycle assay staining using exact same gating parameters for sub-G_1_ cellular fraction indicative of apoptosis. **E,** Transwell cell migration assay. Fluorescence photomicrographs show calcein-stained migrated cells of sgBDNF-AS, sgGMDS-AS1, and sgXLOC_030781 knocked-down 501mel-dCas9-KRAB cells vs. sgROSA negative control and quantification of average sum count cell numbers (*n* = 2).

First, we assessed the cell cycle distribution in response to CRISPRi of lncRNAs BDNF-AS, GMDS-AS1, and also XLOC_030781 in comparison with sgROSA control by measuring the DNA content with 7-AAD dye in vital 501mel [Fig. 3A (top left)] and WM1361 cells [Fig. 3A (bottom left)] using flow cytometry of double GFP-sgRNA–positive and mCherry-Cas9–positive cells [Fig. 3A (left)]. Indeed, the quantification of DNA content assigned to G_1_, G_2_, and S phases [Fig. 3A (right) and B] in 501mel (top) indicated a substantial and significant (BDNF-AS *P* value = 0.0164, GMDS-AS1 *P* value = 0.0075, and XLOC_030781 = 0.0477) G_1_ phase arrest in >70% of cells by knockdown of all three lncRNAs compared with sgROSA control (only ∼50% in the G_1_ phase), which is congruent with a reduced G_2_ phase of about 40% in control to 14% to 20% of cells under lncRNA knockdown. A similar trend for all three lncRNAs although not significant could be observed in the WM1361a cell line (bottom).

We went on to determine the apoptosis rate upon lncRNA knockdown by examining the cleaved and non-cleaved PARP protein levels by immunoblotting of released caspases from mitochondria as a hallmark of apoptosis (Fig. 3C; Supplementary Fig. S4A–S4D; other replicate, densitometric quantification, and qPCR knockdown confirmation; ref. 47). sgROSA served as negative and etoposide apoptosis inducer as positive control in 501mel and WM1361a. In line with the screening results and cell cycle assay, we were able to detect a dramatic increase of cleaved PARP in both cell lines for all three lncRNAs BDNF-AS, GMDS-AS1, and also XLOC_030781, illustrating the importance of their expression in cell survival. We further confirmed this apoptosis phenotype for all lncRNA candidates and in both cell lines by re-analyzing the cell cycle assay 7-AAD flow cytometry using the exact same gating parameters but extrapolating the sub-G_1_ cellular fraction indicative of fragmented DNA and apoptosis (Fig. 3D; ref. 48).

Ultimately, melanoma lethality predominantly arises from the occurrence of metastasis, in which the extensive cell dissemination plays a major role (2). As a simple *in vitro* approximation, a transwell migration assay is considered to reflect the migration and invasion of cells. We performed this assay under lncRNA knockdown condition for BDNF-AS, GMDS-AS1, and XLOC_030781 using the best performing sgRNAs in 501mel cells. Interestingly, only knockdown of XLOC_030781 could attain a robust reduction of transwell migrating cells by ca. 60% (Fig. 3E) eventually in line with its high overexpression found in brain metastasis and LN STCs (Fig. 2F). As no difference in the migrative phenotype was seen for GMDS-AS1 and BDNF-AS repression, we performed another trans-well assay replicate only for XLOC_030781. Likewise, a strong reduction in cell mobility was confirmed for sgXLOC_030781 compared with control (Supplementary Fig. S4E and S4F). Furthermore, we tried to reverse this anti-migrative phenotype by CRISPR-activating BDNF-AS and XLOC_030781 in 501mel. Even though strong expression upregulation for both lncRNAs with each three different sgRNAs ranging between several 10- to up to hundred-fold was accomplished, no obvious difference in the transwell assay was observed, indicating that our assayed phenotype by knockdown alone might not be sufficient (Supplementary Fig. S3G–S3I).

In sum, we phenotypically characterized three CRISPRi lncRNA screening hits and their importance in cell survival and invasive potential *in vitro*.

### XLOC_030781 CRISPRi transcriptome profiling

After having established that our CRISPRi screen resulted in valid lncRNA hits contributing to cell growth, cell cycle apoptosis, and migration in melanoma, we decided to classify deeper the molecular consequences on XLOC_030781 expression at the transcriptomic level. We selected XLOC_030781 for several reasons: (i). Fig. 2F shows upregulation both in brain metastasis and LN STCs melanoma, reflecting unique features eventually relevant for site-independent metastatic lesion formation, (ii) we verify XLOC_030781 lncRNA contributing to cell growth (Figs. 2E, 3A, and B) and survival (Fig. 3C), and (iii) we established an *in vitro* invasive-like phenotype in melanoma (Fig. 3E), outstanding the two other lncRNAs tested. (iv). At the time of the study, XLOC_030781 was a novel lncRNA, which was interesting to study.

**Figure 4:**
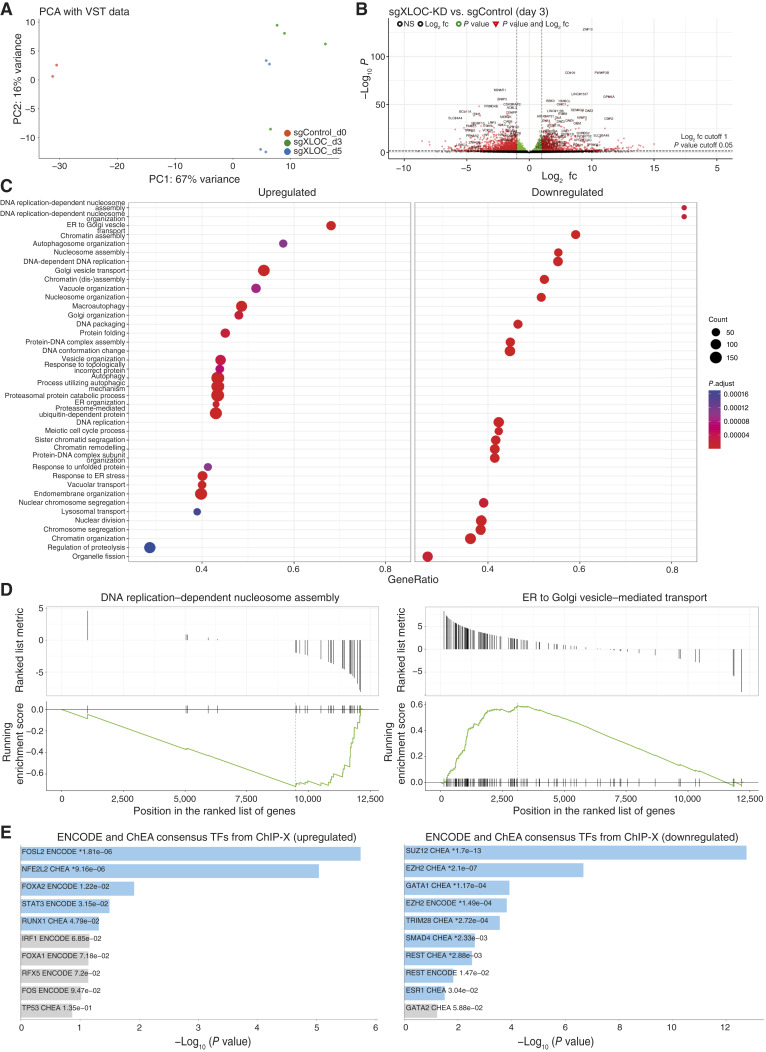
XLOC_030781 CRISPRi knockdown and RNA-seq transcriptomic expression analysis in 501mel-dCas9-KRAB. **A,** Principal component analysis (PCA) of sgXLOC_030781 CRISPRi at days 3 and 5 after infection vs. day 0 negative control (*n* = 2). **B,** Volcano plot of significantly differentially expressed genes (red) upon sgXLOC_030781 knockdown at day 3; *P* value cutoff = 0.05 and log_2_ fc = 1. **C,** GO enrichment analysis of up- and downregulated gene sets upon sgXLOC_030781 knockdown at day 3. **D,** GSEA enrichment analysis of each top example of upregulated (ER to Golgi vesicle transport, left) and downregulated genes (DNA replication–dependent nucleosome assembly, right). **E,** Upstream TF analysis of up- and downregulated genes using ChEA upon sgXLOC_030781 knockdown at day 3.

To perturbate XLOC_030781 lncRNA expression, the most effective sgRNAs (sgRNA6 and sgRNA7) were employed each in technical duplicates including two negative control samples in 501-mel-tetON dCas9 KRAB cells Dox treated for 3 and 5 days. Ribo-depleted RNA-converted cDNA was then subjected to deep sequencing and differential transcriptome analysis. Principal component analysis showed well separation of all sgXLOC_030781 treated against control samples at both time points on PC1 explaining 67% variance (Fig. 4A). DGE analysis showed a dramatic and significant transcriptome divergence upon lncRNA knockdown compared with control at both time points (Fig. 4B; Supplementary Fig. S5A log_2_ fc = 1, *P* value 0.05; Supplementary Table S4), whereas there were only marginal expression differences between sgRNA knockdowns at days 3 and 5 (Supplementary Fig. S5B). In total, we observed 1,294 genes up- and 1,462 downregulated at day 3 and 986 up- and 1,179 downregulated at day 5. Subsequent GO term enrichment analysis of differentially expressed genes (Fig. 4C, top 20 GO terms up- and downregulated genes) at day 3 highlighted features such as DNA replication–dependent nucleosome assembly and DNA replication–dependent nucleosome organization in the downregulated genes. This might implicate XLOC_030781 functioning in cell division and proliferation at the transcriptional or epigenetic level. In the upregulated genes, GO terms like endoplasmic reticulum (ER) to Golgi vesicle–mediated transport or autophagosome organization appeared [also shown in gene set enrichment analysis (GSEA) plots in Fig. 4D]. Of note, GO terms of upregulated genes were quite disperse compared with the downregulated gene set, with the first ones mainly consisting of cell functions located in the cytoplasm whereas latter one’s functions take place predominantly in the nucleus. In general, the same pathways and GSEA at day 5 resembled well the ones on day 3 and are summarized in Supplementary Fig. S5C and S5D, confirming our data.

We next aimed to delineate potential molecular functions of XLOC_030781 from gene signatures obtained by our knockdown RNA-seq approach. Recently, it has been proposed that certain lncRNAs functionally act to cis-regulate their neighbor gene expression by shaping the proximal chromatin environment (5). To check this hypothesis, we collected the expressed 14 neighbor genes of a window approximately one megabase up- or downstream of the XLOC_030781 locus and plotted the *z*-score–normalized gene expression of sgRNA-treated vs. control samples at all time points. We found no significant expression correlation between those comparisons ruling out the possibility that XLOC_030781 serves as a cis-regulator at the neighboring chromatin (Supplementary Fig. S5E). We then meta-analyzed the enrichment of differentially expressed genes for their occupancy and regulation by different upstream TEs using ENCODE and ChEA consensus TFs from ChIP-X, a TF target library relying on deposited ChIP-seq data (33). Top two *P* value–ranked enriched TFs among the XLOC_030781 knocked down genes included FOSL2 and NFE2L2 TFs and among the upregulated genes, SUZ12 and EZH2, both components of the PRC2 repressor complex (49). Although all these TFs are already associated also as drivers of melanoma (50–54), their exact implications in the XLOC_030781 phenotype drivers remain inconclusive. Supplementary Fig. S5F is presenting representative visualized track information about the identified H3K4me3 and H3K27ac ChIP-seq peaks near the XLOC_030781 locus transcription start site in melanocytes, brain metastasis, LN, and WM1361a cell line.

As an extraordinary high number of genes were misregulated upon XLOC_030781 knockdown, we asked the question whether this might be an effect of impaired DNA damage repair or reduced genome stability. The Comet assay is a single-cell approach to quantify affected DNA damage repair and DNA fragmentation accumulation. We performed this assay upon sgXLOC_030781 condition at two different time points at days 3 and 5 after sgRNA infection in 501mel and WM1361a cells in duplicates. Nontreated cells served as negative control and DNA-oxidizing agent H_2_O_2_ treated cells as positive control. Tail-generating cells were quantified by microscopy and 501mel treated with sgRNA showed a significant higher proportion of cells with a Comet (>20%, *P* value = 0.0076, Student *t* test, two-paired) at day 3 as compared with control (ca. 5%). All the other time points and cell lines showed a similar trend; No significant difference could be observed (Supplementary Fig. S5G and S5H). This indicates that XLOC_030781 might be involved in genome stability and DNA and damage repair.

### TCGA data mining of XLOC_030781/ENSG00000287723

**Figure 5:**
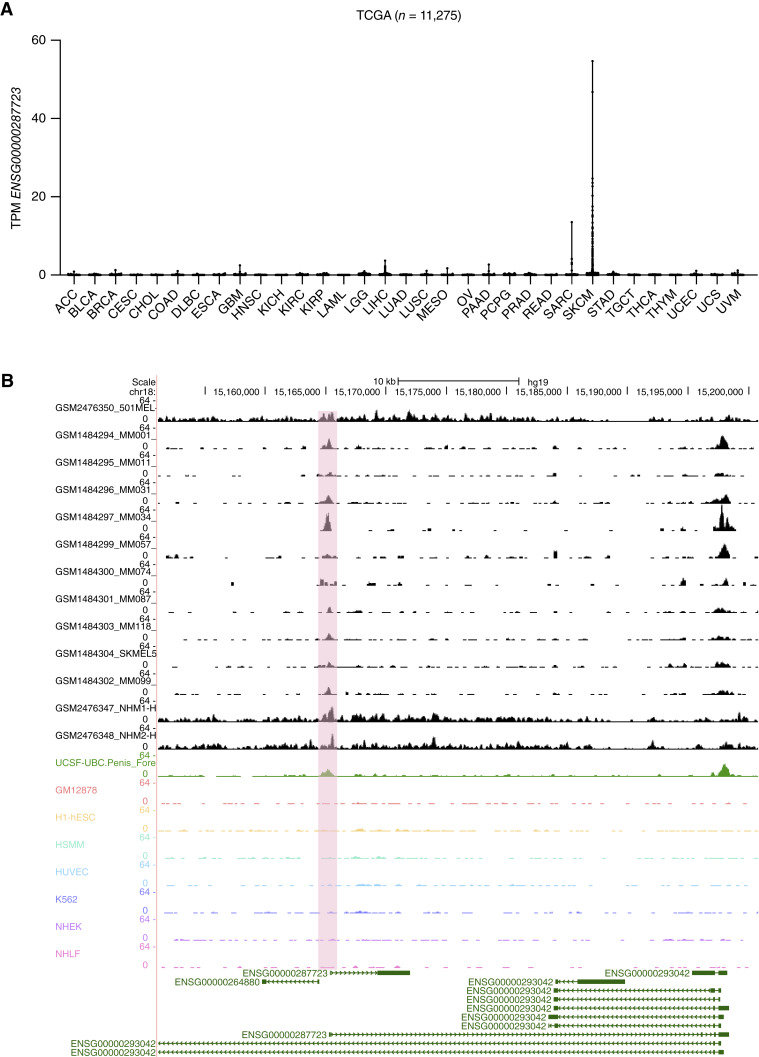
TCGA XLOC_030781 expression data analysis in cancer and clinical melanoma samples. **A,** Pan-cancer expression analysis using TCGA. SKCM, skin cutaneous melanoma. **B,** UCSC genome browser snap shots of H3K27ac ChIP-seq data from melanoma (MM, black), non-malignant skin biopsies (NHM, black), foreskin sample (green), and other malignant samples (other colors) at the XLOC_030781/ENSG00000287723 locus. Pink bar shows the peak area of interest in the vicinity of XLOC_030781 promoter. ACC, adrenocortical carcinoma; BLCA, bladder urothelial carcinoma; BRCA, breast invasive carcinoma; CESC, cervical squamous cell carcinoma and endocervical adenocarcinoma; CHOL, cholangiocarcinoma; COAD, colon adenocarcinoma; DLBC, diffuse Large B-cell Lymphoma; ESCA, esophageal carcinoma; GBM, glioblastoma multiforme; HNSC, head and neck squamous cell carcinoma; KICH, kidney Chromophobe; KIRC, kidney renal clear cell carcinoma; KIRP, kidney renal papillary cell carcinoma; LAML, acute myeloid leukemia; LGG, low grade glioma; LIHC, liver hepatocellular carcinoma; LUAD, lung adenocarcinoma; LUSC, lung squamous cell carcinoma; MESO, mesothelioma; OV, ovarian serous cystadenocarcinoma; PAAD, pancreatic adenocarcinoma; PCPG, pheochromocytoma and paraganglioma; PRAD, prostate adenocarcinoma; READ, rectum adenocarcinoma; SARC, sarcoma; SKCM, skin cutaneous melanoma; STAD, stomach adenocarcinoma; TGCT, testicular germ cell tumors; THCA, thyroid carcinoma; THYM, thymoma; UCEC, uterine corpus endometrial carcinoma; UCS, uterine carcinosarcoma; UVM, uveal melanoma.

During the course of the article preparation, it turned out that lncRNA XLOC_030781 became annotated as ENSG00000287723. We therefore were able to mine clinically relevant TCGA expression datasets for XLOC_030781 and clinical associations thereof. First, we profiled XLOC_030781 expression in 11,275 cancer samples over 33 tumor entities and found this lncRNA to be very skin cutaneous melanoma specific with a minor additional expression signature present in sarcoma (Fig. 5A). We also verified a relatively skin cancer–specific expression pattern by re-analyzing public H3K27ac enhancer–promoter mark ChIP-seq data from the UCSC genome browser in several melanoma and non-melanoma samples (Fig. 5B). In line with this notion, no H3K27ac peak could be detected among the promoter region in cells of lymphoblast, embryonic stem cell, erythroid, endothelial, epidermal, or fibroblast origin. This could indicate that XLOC_030781 could be a melanoma-specific lncRNA involved in growth and progression to be targeted.

Lastly, we exploited two published datasets from (34) to (35) in order to stratify XLOC_030781 expression in primary melanoma (*n* = 51 Badal and colleagues and *n* = 57 Kunz and colleagues; Supplementary Table S6) and nevi biopsies (*n* = 27 Badal and colleagues and *n* = 23 Kunz and colleagues; Supplementary Table S6; Supplementary Fig. S6A). Unfortunately, in neither of these groups a significant expression difference could be found nor in a larger TCGA data analysis of skin cutaneous melanoma metastatic lesions (*n* = 367) in comparison with 76 primary melanomas (Supplementary Fig. S6B). Additionally, high expression of XLOC_030781 seemed to be associated with a significantly higher patient survival probability (*P* value = 0.0216) as compared with lowly expressing patients with melanoma (Supplementary Fig. S6C). This might be attributed to the fact that we screened lncRNAs based on their survival phenotype but also because of pleiotropic and strong context-dependent functions generally found among lncRNAs (39, 40). However, a direct comparison of melanocytes versus metastasis as done by us has not been published in a larger sample set, omitting direct conclusions.

To better understand biological features of patients carrying high and low expression of XLOC_030781 (Supplementary Fig. S6D), we performed DESeq2 analysis on a subset of TCGA patients with melanoma (high = 20 and low = 125) and found 3,656 and 4,458 genes significantly associated to high (XLOC^High^) and low (XLOC^Low^) expression of XLOC_030781, respectively (adj *P* val. < 0.05; Supplementary Fig. S6E and S6F). GO analysis of these genes showed a significant association with neural-like, angiogenesis, and calcium channel genes in XLOC^High^ patients. On the contrary, XLOC^Low^ patients expressed genes involved in keratinocyte biology and pigmentation (i.e., *MITF*, *TYR*, and *TYRP1*; Supplementary Table S7).

## Discussion

The current study examines the expression of lncRNAs in metastatic melanoma derivatives compared with healthy cells, as well as in various melanoma cell lines. The goal is to lay the foundation for a focused CRISPR inhibition screening of lncRNAs involved in melanoma progression. Our hypothesis was that some of these lncRNAs may have cancer cell fitness–promoting or growth-promoting properties, potentially increasing the chances of identifying new ones associated with metastatic melanoma while minimizing screening efforts. Through this approach, we identified thousands of expressed lncRNAs, including many novel ones. Among these, 262 were significantly overexpressed, significantly expanding the known repertoire of lncRNAs associated with metastatic melanoma (55), potentially holding relevance for disease pathology. However, we excluded an in-depth study of lncRNAs upregulated in BRAFi/MEKi-resistant background, although potentially being cell growth or survival relevant, but more important in current second-line treatment regimens.

In the light of metastatic melanoma as a major factor of fatality in patients, we anticipated a positive disease-progressing or metastatic effect (2) of specifically upregulated lncRNAs, which also may be easily amendable to malignant targeting other than tumor suppressor lncRNAs. Additionally, we also consider highly but not differentially expressed lncRNAs as potentially valuable screening targets as they can also affect melanoma progression and those ones are missed by our approach. However, those lncRNAs would not serve as attractive novel molecular targets as they cannot distinguish between pathologic and healthy cells. Beyond proliferation and invasion, pro-tumorigenic traits include oncogenic activation, immune evasion, microenvironment remodeling, and epigenetic changes. Although lncRNAs likely influence these processes, their characterization requires extensive screening beyond our study's scope. Another limitation of the current study is that it relied solely on genetic knockdown to perturb genes potentially involved in melanoma fitness and survival to detect genetic dependencies (56, 57) but not e.g., overexpressed oncogenic lncRNAs. *Bona fide* oncogenes, which can *per se* initiate tumor progression by gain of function as a result of activating mutations or overexpression, are missed by this approach. However, artificial overexpression may not reflect endogenous regulatory mechanisms, can be even detrimental to disturb well-adjusted molecular stoichiometries, or can induce secondary effects unrelated to the gene’s primary function. Moreover, oncogenes often function in a context-dependent manner, which overexpression studies fail to capture (58).

With our approach, we successfully validated the impaired growth phenotype of four of the top 10 hits from the screening (GMDS-AS1, BDNF-AS, RP11-267N12.3, and XLOC_030781). Three of these were further characterized, demonstrating their positive impact on cell cycle regulation, apoptosis, and invasion *in vitro*. Our primary candidate, XLOC_030781, underwent additional analysis, revealing significant transcriptional changes upon perturbation. Its affection of GO terms related to nucleosome assembly and vesicle transport suggest bi- or multicellular functions in melanoma cells. The final candidate lncRNAs studied in more detail were BDNF-AS, GMDS-AS1, and XLOC_030781 that ranked highly (among the top 10) in both CRISPRi screens: BDNF-AS is located on chromosome 11 (genomic coordinates from GRCh38.p14 assembly; chromosome 11: 27,506,830-27,698,231 forward strand) and has been implicated in various cancer types. Interestingly, although it is consistently downregulated across cancers like colorectal cancer, esophageal cancer, cervical cancer, glioblastoma, and osteosarcoma (59), its role in melanoma remains unexplored. On the other hand, GMDS-AS1 is a novel lncRNA transcribed from a genomic locus on chromosome 6 (genomic coordinates from GRCh38.p14 assembly; chromosome 6: 2,245,718-2,525,976 forward strand). To date, studies have shown its antitumorigenic properties in lung adenocarcinoma and hepatocellular carcinoma while promoting tumorigenesis in colorectal cancer (60–62). Similar to BDNF-AS, its association with melanoma has not been explored until now. Our findings suggest that both BDNF-AS and GMDS-AS1 affect cell-cycle progression and apoptosis but not migration. This indicates a role in cell survival rather than metastasis.

Lastly, XLOC_030781 represents an unannotated lncRNA located on chromosome 18 (genomic coordinates from GRCh38.p14 assembly; chromosome 18: 15,164,633-15,164,933). Designated as ENSG00000287723 since September 2023, this lncRNA remains largely unexplored, with existing information being limited to its expression in various cell types and tissues, including the cortical plate, ventricular zone, ganglionic eminence, and 68 other cell types or tissues (63). However, among the 33 analyzed cancer types, XLOC_030781 seems to exhibit a lncRNA expression pattern relatively specific to melanoma, suggesting a particular function in this context, which may be potentially interesting for selectively targeting growth.

A major caveat in studying the migratory capacity of XLOC_030781 *in vitro* is that we established a phenotype solely upon knockdown (Fig. 3E). This suggests that the lncRNA may be required for the observed effects; however, its sufficiency remains unconfirmed as overexpression did not produce a detectable inverse phenotype (Supplementary Fig. S4G and S4I). Also, the migratory capacity observed at least for *in vitro* knockdown for XLOC_030781 cannot be easily translated to *in vivo* models and further clarification is needed about their metastatic phenotype in patients. Additionally, we show significant upregulation of gene sets involved in ER to golgi vesicle transport *in vitro*, implying that the general cell surface protein presentation may be affected upon repression of XLOC_030781 (Fig. 4C). This process is critical for the immunogenicity of tumor cells and this effect of XLOC_030781 could potentially influence HLA surface expression and recognition by the host immune system (64). The high genetic mutational burden together with the hypermetabolic environment found in malignant melanoma leads to massive disturbance of proteolysis and, in consequence, to ER stress or unfolded protein response. All these functional features we have identified to be increased upon XLOC_030781 knockdown in our RNA-seq data [Fig. 4C (left)]. ER stress can trigger cell fate toward a pro-survival or pro-apoptotic phenotype depending on the signal duration and intensity. As XLOC_030781 knockdown suppresses both cell-cycle progression and decreases apoptosis, it can be assumed that it plays a significant role in fine balancing the outcomes toward cell survival eventually including resistance against autophagy and cell death, another functional GO term which is associated with XLOC_03081 knockdown (65). Yet, uncovering the exact mechanisms and determining whether these phenotypes are relevant *in vivo* or *in situ* remain unclear, necessitating further studies in preclinical models for clarification.

Moreover, the substantial number of differentially expressed genes in our RNA-seq analysis of XLOC_030781 raises intriguing questions about its underlying principle. One possibility is that XLOC_030781 orchestrates the function of critical master regulators in transcription or acts on epigenetic regulation. However, our TF analysis (see Fig. 4E) did not provide a clear answer to this. Alternatively, XLOC_030781 may safeguard genomic integrity as suggested by GSEA of downregulated transcripts related to DNA-dependent replication or nucleosome assembly (Supplementary Fig. S4C and S5C), along with our Comet assay findings, (Supplementary Fig. S5F) which may point toward the latter direction (66–68). However, extensive genomic, biophysical, and *in vivo* work is required in the future to holistically understand the multifunctional roles of XLOC_030781 and its implications for melanoma progression and pathogenesis.

With this study, we enlarge the compendium of novel and potentially critical lncRNAs in melanoma. We intensively studied three lncRNA examples with implications on cell survival, growth, and migration. However, our work significantly lacks *in vivo* confirmation related to invasiveness and primary tumor formation as well as potential preclinical treatment modalities via application e.g., with anti-sense oligonucleotides especially in an immunocompetent humanized background to address the discrepancy between patient survival chance and *in vitro* phenotype. This work could be also complemented by an extended set of samples from a multi-institutional cohort to validate our TCGA datamining.

In conclusion, we provide a powerful set of potentially relevant and novel lncRNAs in melanoma although the usefulness of some of the identified lncRNAs as biomarkers in disease or even future therapeutic target structures remains to be determined.

## Supplementary Material

Figure S1 IGV browser snapshot histone marks at MITF-SAMMSON locus

Figure S2 CRISPRi system establishment in melanoma cell lines

Figure S3 LncRNA CRISPRi screen and controls in melanoma cells

Figure S4 Apoptosis and lncRNA OE transmigration assays

Figure S5 XLOC_030781 CRISPRi knockdown and RNA-sequencing transcriptomic expression analysis

Figure S6 TCGA metaanalysis of lncRNA XLOC_030781

Table S1 lncRNA regulatory programs

Table S2 Fifty non-targeting sgRNAs were incorporated as negative controls as well as positive controls against five known melanoma-associated lncRNAs.

Table S3 CRISPRi screening results

Table S4 Results XLOC_030781 KD RNA-Seq

Table S5 List used Oligonucleotides

Table S6 Samples and expression values Badal et al and Kunz et al
