# Supplementary material for: Uncovering Novel lncRNAs Linked to Melanoma Growth and Migration with CRISPR Inhibition Screening
Source: Cancer Res Commun. 2025 Jul 9;5(7):1102–18. doi: 10.1158/2767-9764.CRC-24-0416 (PMC12238846; doi:10.1158/2767-9764.CRC-24-0416)
Supplement: Figure S1 — IGV browser snapshot histone marks at MITF-SAMMSON locus [file crc-24-0416_figure_s1_suppsf1.pdf]

Figure S1

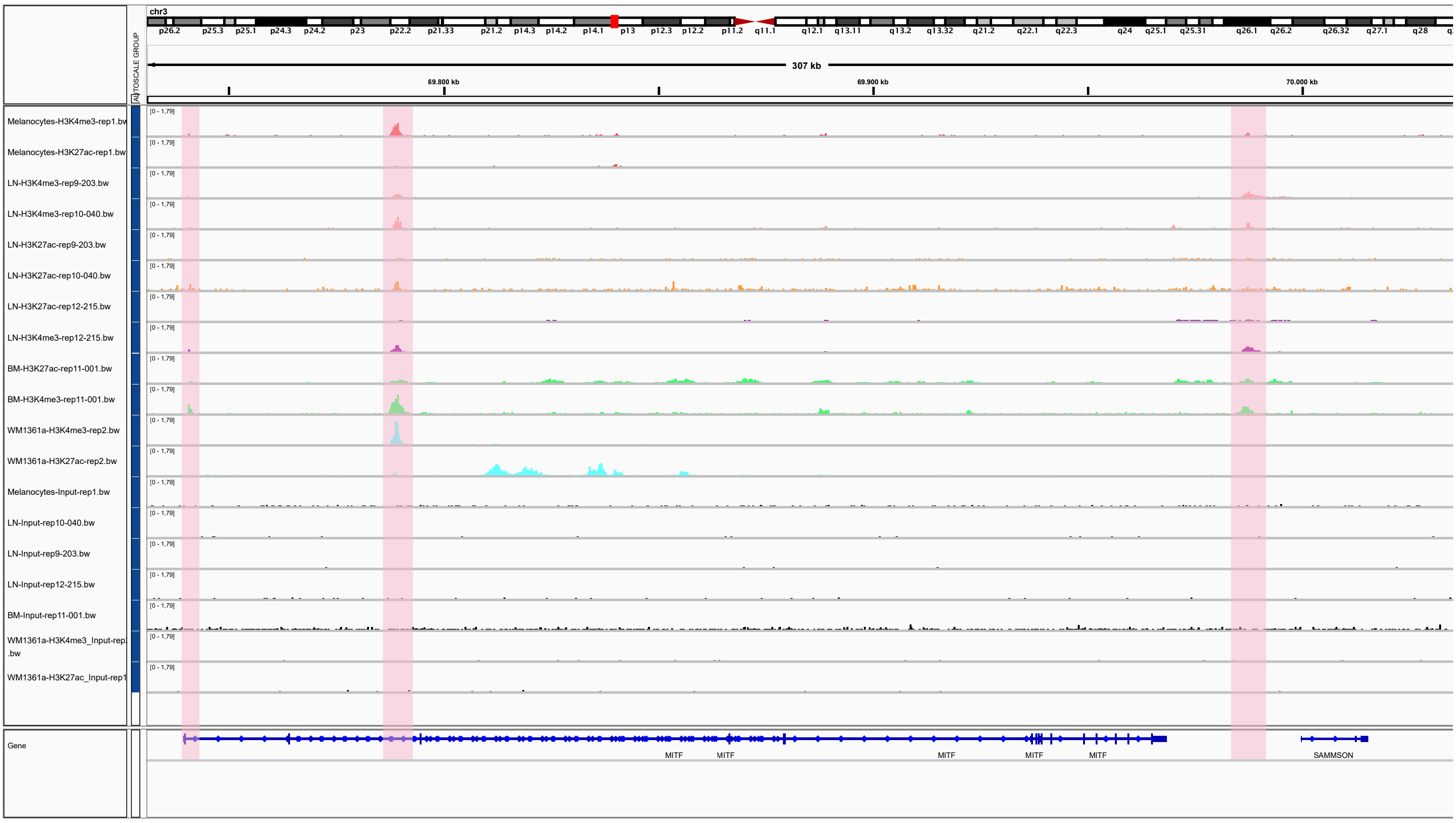

Figure S1:
IGV browser snapshot of ChIP-Seq analysis for H3K4me3 and H3K27ac and input control of the MITF-SAMMSON co-locus for representative melanocytes, BM, LN and WM1361a. Red boxes indicate identified peaks near the respective TSS.
