# Supplementary material for: Uncovering Novel lncRNAs Linked to Melanoma Growth and Migration with CRISPR Inhibition Screening
Source: Cancer Res Commun. 2025 Jul 9;5(7):1102–18. doi: 10.1158/2767-9764.CRC-24-0416 (PMC12238846; doi:10.1158/2767-9764.CRC-24-0416)
Supplement: Figure S2 — CRISPRi system establishment in melanoma cell lines [file crc-24-0416_figure_s2_suppsf2.pdf]

Figure S2

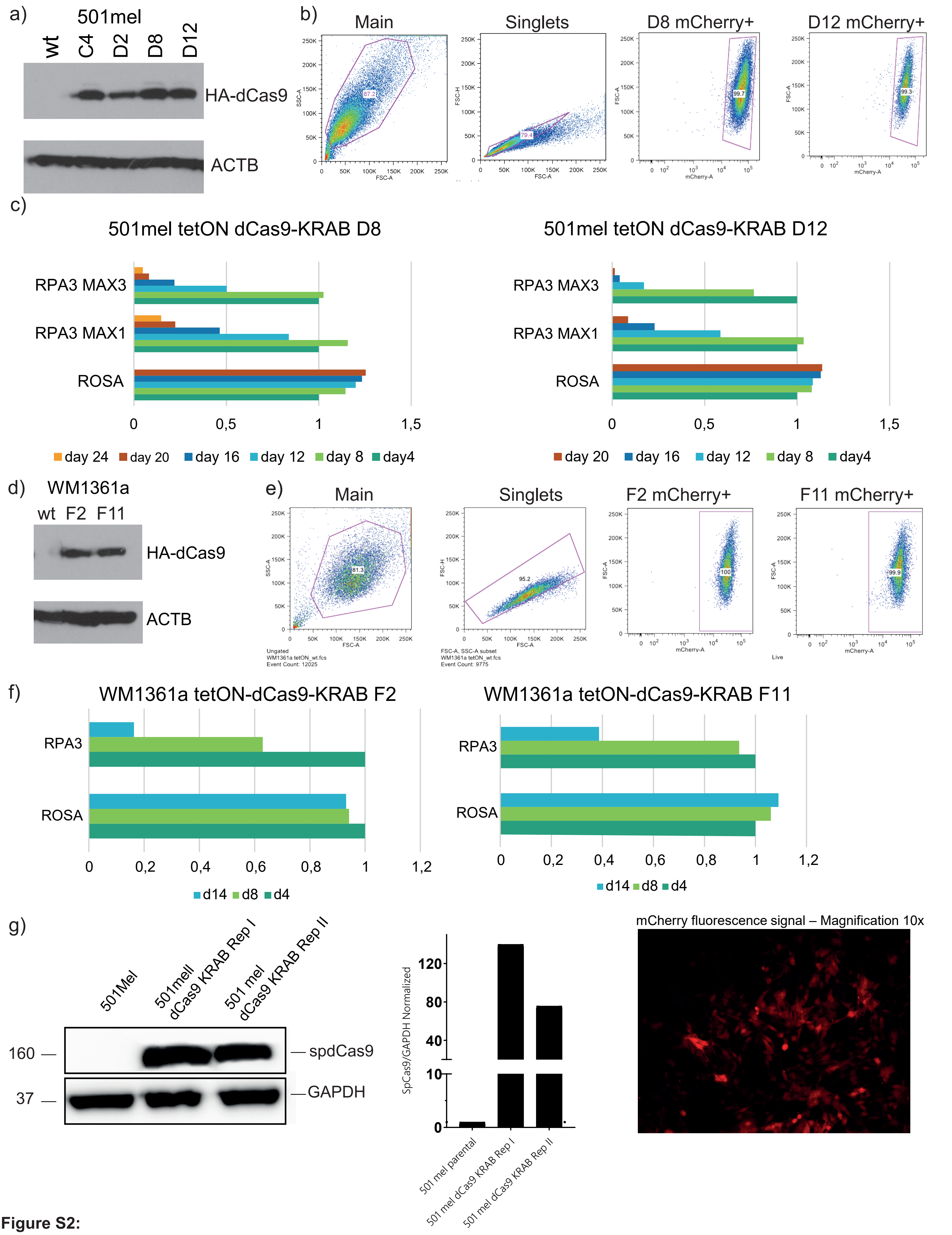

Figure S2:
CRISPRi system establishment in melanoma cell lines. HA-tagged dCas9-KRAB protein expression in diverse single cell clones by Western-blot, mCherry-dCas9-KRAB by flow-cytometry and GFP competition assays for 2 sgRNAs targeting essential gene RPA1 and sgROSA negative control over indicated time day 4 to day 20 in a)- c) 501mel single clones D8 and D12 and d)-f) WM1361a F2 and F11 over day 4 to day 14. g) dCas9-KRAB expression confirmation in 501mel cell pool by Western-blot and mCherry fluorescence detection for repetition of CRISPRi screen 2.0. (related to supplemental Figure S3).
