# Supplementary material for: Uncovering Novel lncRNAs Linked to Melanoma Growth and Migration with CRISPR Inhibition Screening
Source: Cancer Res Commun. 2025 Jul 9;5(7):1102–18. doi: 10.1158/2767-9764.CRC-24-0416 (PMC12238846; doi:10.1158/2767-9764.CRC-24-0416)
Supplement: Figure S3 — LncRNA CRISPRi screen and controls in melanoma cells [file crc-24-0416_figure_s3_suppsf3.pdf]

Figure S3

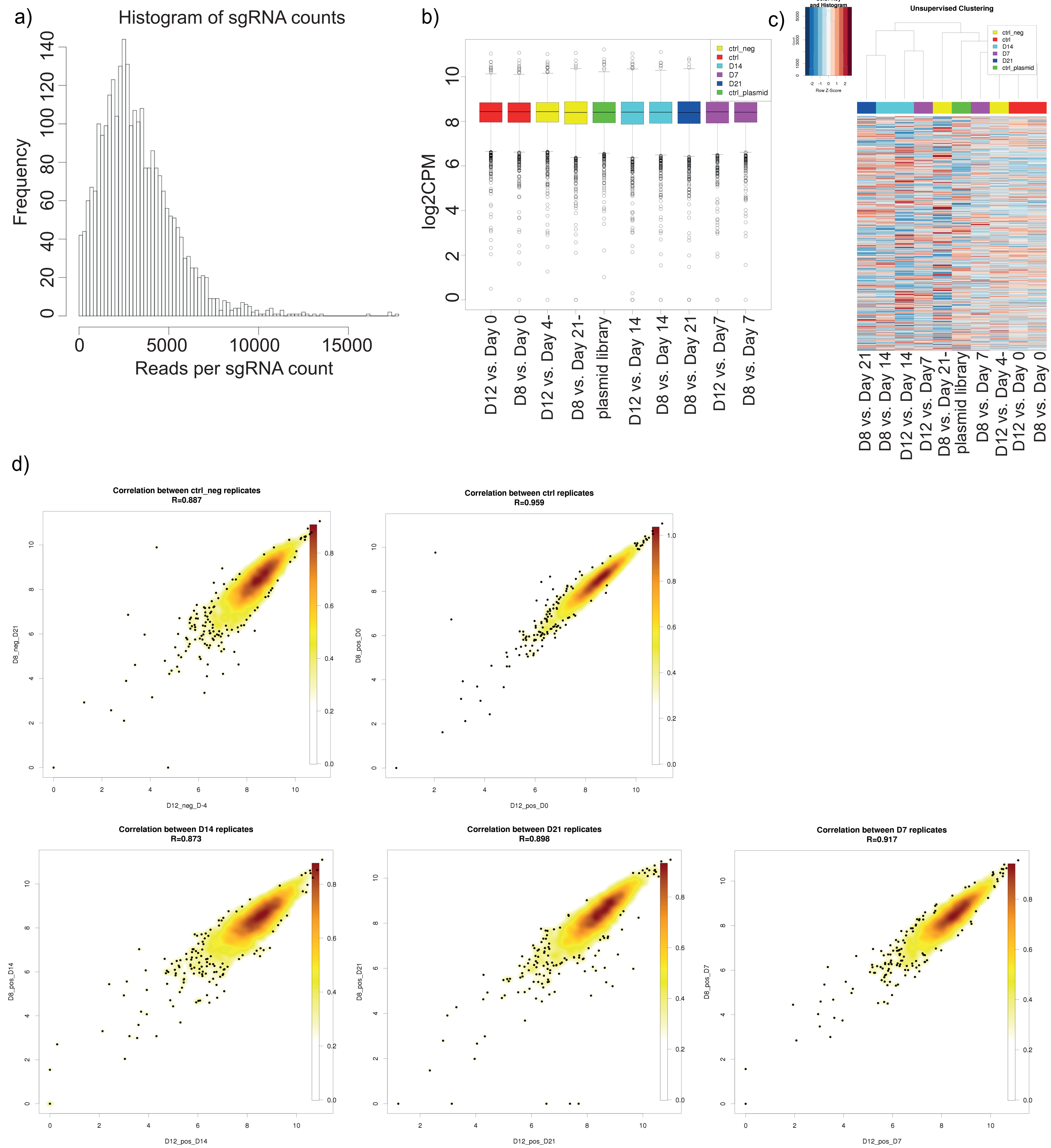
a)
Histogram of sgRNA counts
Frequency
Reads per sgRNA count
b)
log2CPM
ctrl_neg
ctrl
D14
D7
D21
ctrl_plasmid
D12 vs. Day 0
D8 vs. Day 0
D12 vs. Day 4-
D8 vs. Day 21-
plasmid library
D12 vs. Day 14
D8 vs. Day 14
D8 vs. Day 21
D12 vs. Day7
D8 vs. Day 7
c)
Color Key and Histogram
Row Z-Score
Unsupervised Clustering
D8 vs. Day 21
D8 vs. Day 14
D12 vs. Day 14
D12 vs. Day7
D8 vs. Day 21-
plasmid library
D8 vs. Day 7
D12 vs. Day 4-
D12 vs. Day 0
D8 vs. Day 0
d)
Correlation between ctrl_neg replicates
R=0.887
D12_neg_D-4
D8_neg_D21
Correlation between ctrl replicates
R=0.959
D12_pos_D0
D8_pos_D0
Correlation between D14 replicates
R=0.873
D12_pos_D14
D8_pos_D14
Correlation between D21 replicates
R=0.898
D12_pos_D21
D8_pos_D21
Correlation between D7 replicates
R=0.917
D12_pos_D7
D8_pos_D7

Figure S3 (continued)

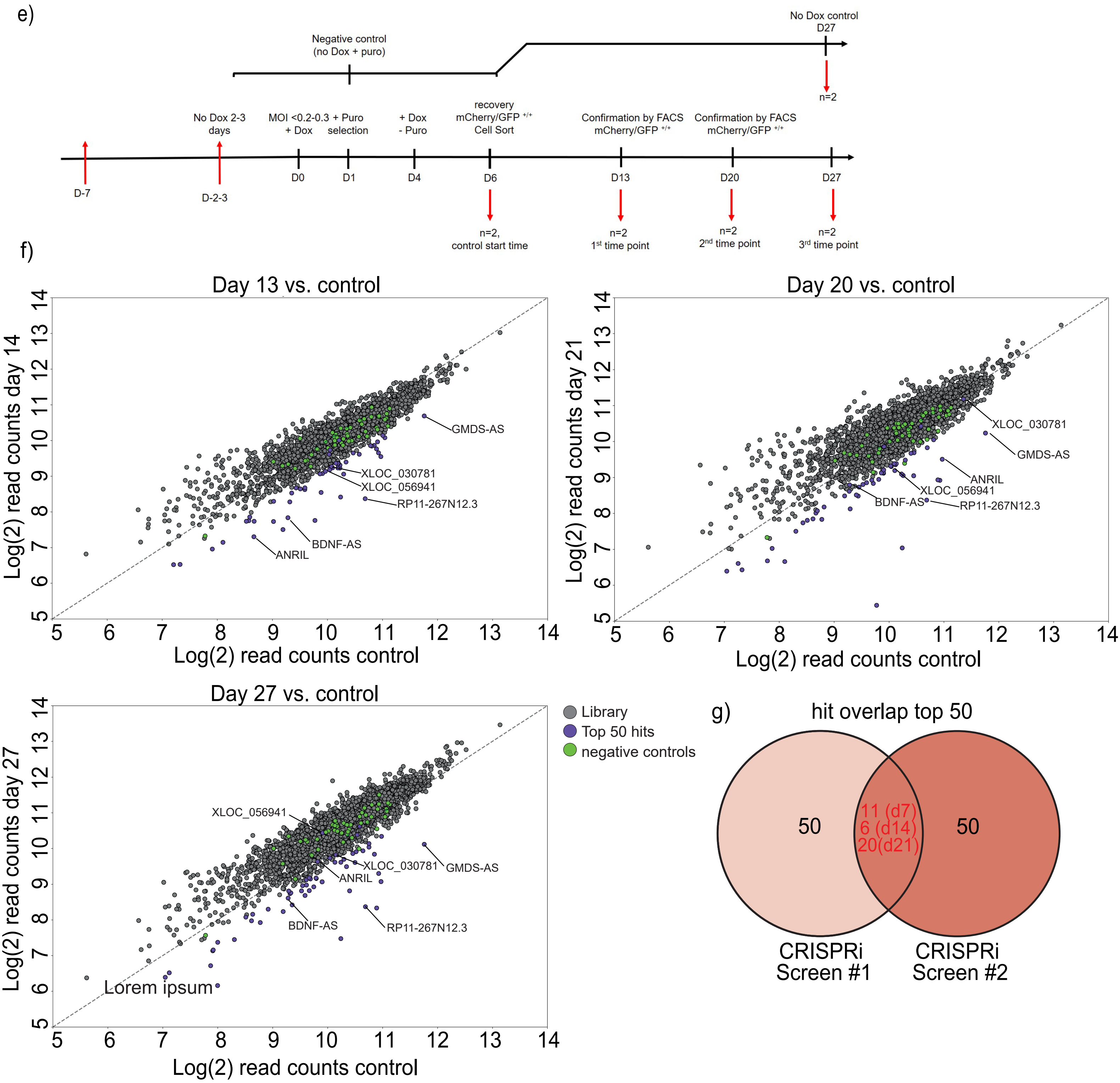

Figure S3:
LncRNA CRISPRi screen in melanoma 501mel cells: a) Histogram shown raw reads of sgRNA frequency distribution of plasmid library. D8 and D12 refers to selected single cell clone of 501mel-dCas9-KRAB and D7, D14 and D21 for the days of screening time. b) Box-plot of log2 cpm sgRNA counts during CRISPRi screen. c) heatmap showing unsupervised hierarchical clustering of z-score normalized sgRNA expression throughout all samples indicated. d) Density dot-plots for lncRNA CRISPRi screen over replicates for correlation (n=2), correlation coefficient is indicated. e) time-line of lncRNA CRISPRi screen 2.0. f) Dot-plots shown results of CRISPRi lncRNA library screen 2.0 at time points day 13, 20 and 27 post-selection start as log2 read counts of control day 0 vs. respective time points. Each dot represents a single sgRNA. Selected lncRNA hits from CRISPRi screen as well as lncRNA positive control ANRIL are highlighted in purple as depleted. Negative control sgRNAs are shown in green. g) Venn-diagram showing the overlap of lncRNA top 50 hits in both screens at all time points.
