# Supplementary material for: Uncovering Novel lncRNAs Linked to Melanoma Growth and Migration with CRISPR Inhibition Screening
Source: Cancer Res Commun. 2025 Jul 9;5(7):1102–18. doi: 10.1158/2767-9764.CRC-24-0416 (PMC12238846; doi:10.1158/2767-9764.CRC-24-0416)
Supplement: Figure S4 — Apoptosis and lncRNA OE transmigration assays [file crc-24-0416_figure_s4_suppsf4.pdf]

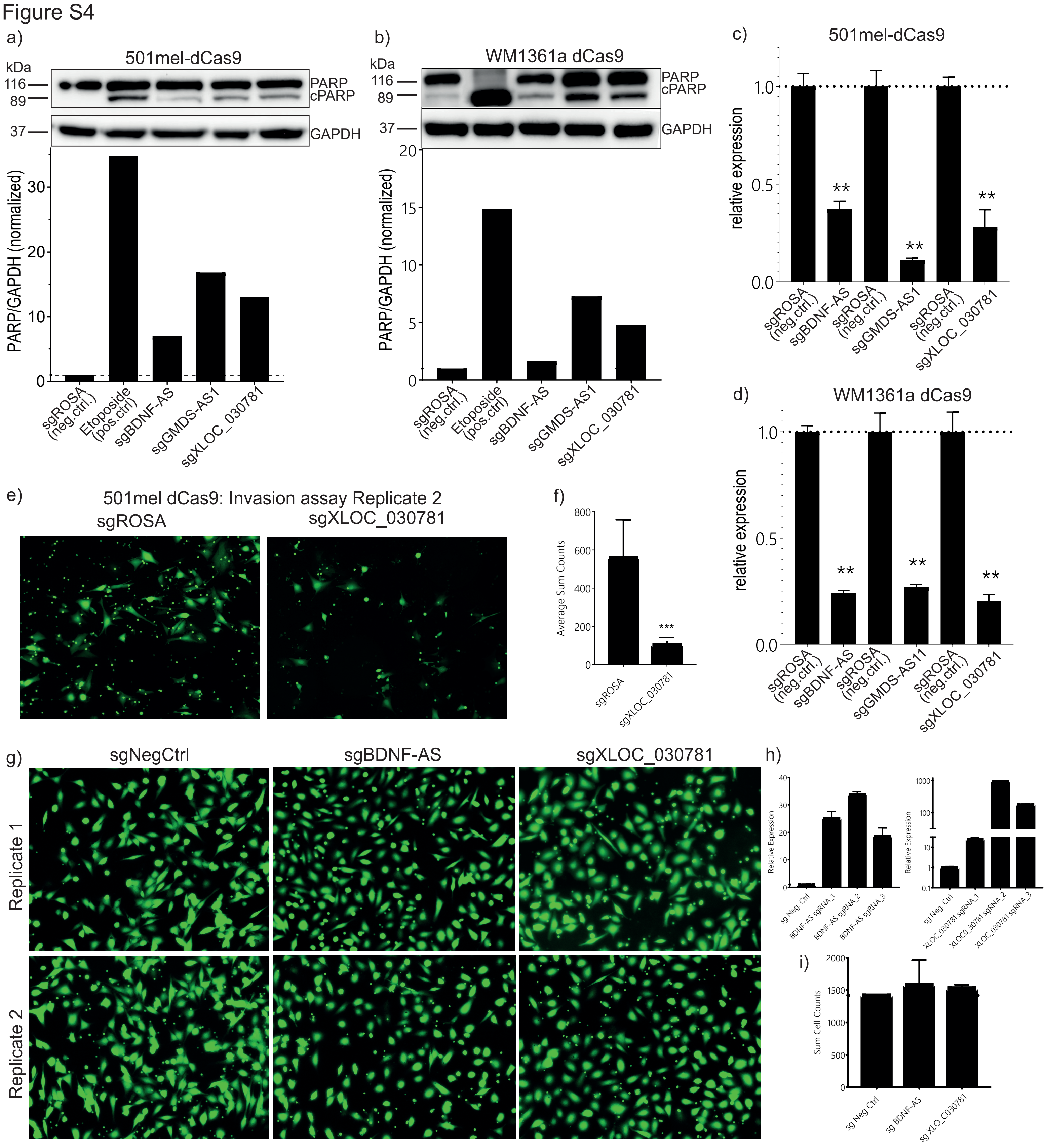

Figure S4:

a) and b) PARP cleavage apoptosis assay replicate 2 by Western-blot and densitometric quantification in 501mel-dCas9-KRAB and WM1361a-dCas9-KRAB (related to Figure 3c). c) and d) qPCR knockdown confirmation of sgBDNF-AS, GMDS-AS1 and XLOC_030781 in 501mel-dCas9-KRAB and WM1361a-dCas9-KRAB relative to GAPDH and sgROSA control. e) Trans-well cell migration assay. Fluorescence photomicrographs show calcein stained migrated cells of sgXLOC_030781 knocked-down 501mel-dCas9-KRAB cells vs. sgROSA negative control and quantification of average sum count cell numbers (replicate 2, related to Figure 3 d) g-i) Trans-well cell migration assay. Fluorescence photomicrographs show calcein stained migrated cells of sgXLOC_030781 and sgBDNF-AS 501mel CRISPR-activation cell cells vs. sgNegative control. h) confirmation of lncRNA sgXLOC_030781 and sgBDNF-AS overexpression by qPCR and i) quantification of average sum count cell numbers.
