# Supplementary material for: Uncovering Novel lncRNAs Linked to Melanoma Growth and Migration with CRISPR Inhibition Screening
Source: Cancer Res Commun. 2025 Jul 9;5(7):1102–18. doi: 10.1158/2767-9764.CRC-24-0416 (PMC12238846; doi:10.1158/2767-9764.CRC-24-0416)
Supplement: Figure S5 — XLOC_030781 CRISPRi knockdown and RNA-sequencing transcriptomic expression analysis [file crc-24-0416_figure_s5_suppsf5.pdf]

Figure S5

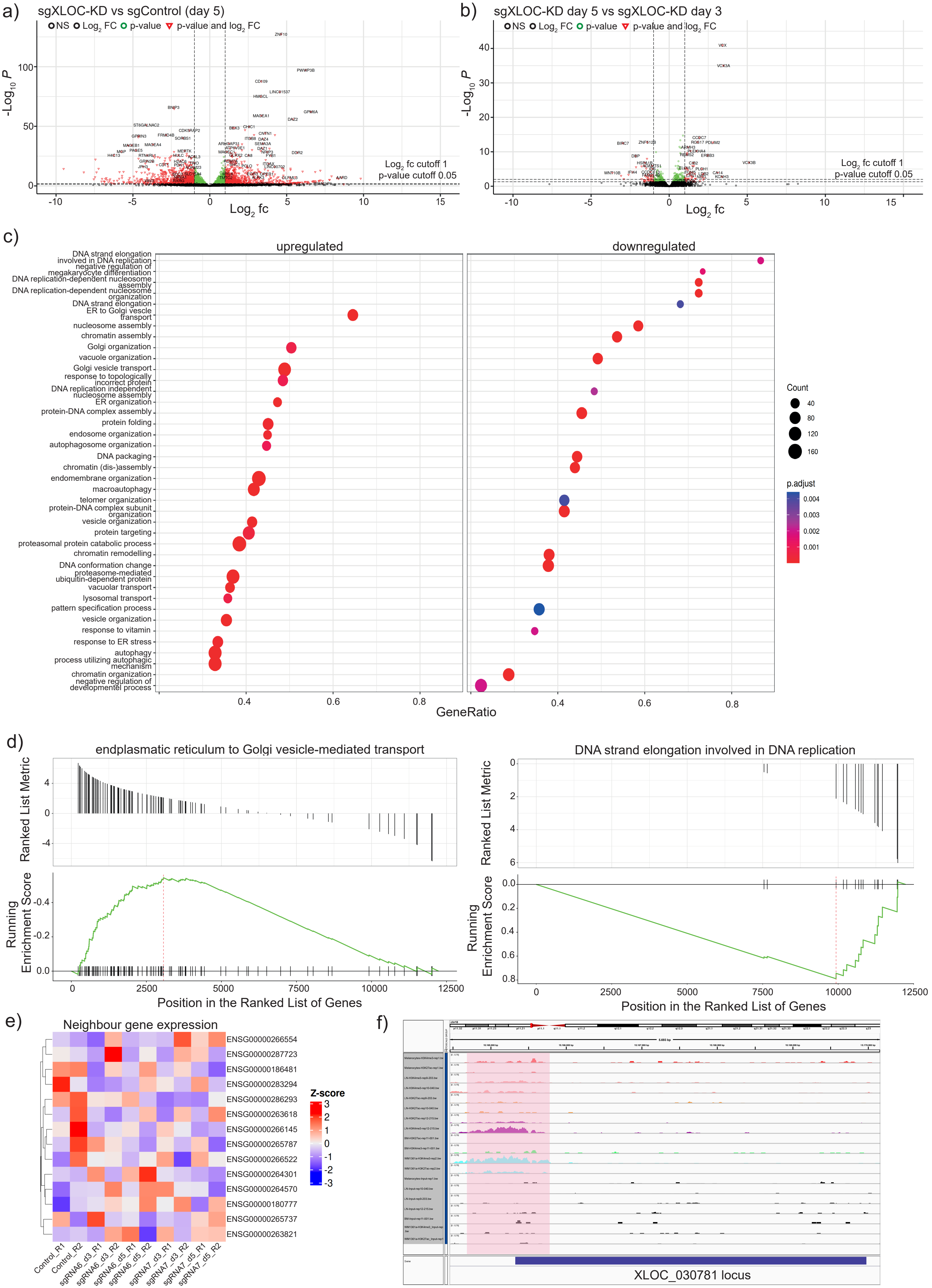

a)
sgXLOC-KD vs sgControl (day 5)
NS
p-value
p-value and log2 FC
Log2 fc cutoff 1
p-value cutoff 0.05
Log2 fc
-Log10 P
ZNF10
PWWP3B
CD109
LINC01537
HMGCL
GPM6A
MAGEA1
DAZ2
BNIP3
ST6GALNAC2
CDK5RAP2
DDR2
b)
sgXLOC-KD day 5 vs sgXLOC-KD day 3
VCX
VCX3A
VCX3B
CCDC7
BIRC7
ZNF612B
Log2 fc cutoff 1
p-value cutoff 0.05
c)
upregulated
downregulated
DNA strand elongation involved in DNA replication
negative regulation of megakaryocyte differentiation
DNA replication-dependent nucleosome assembly
DNA replication-dependent nucleosome organization
DNA strand elongation
ER to Golgi vesicle transport
nucleosome assembly
chromatin assembly
Golgi organization
vacuole organization
Golgi vesicle transport
response to topologically incorrect protein
DNA replication independent nucleosome assembly
ER organization
protein-DNA complex assembly
protein folding
endosome organization
autophagosome organization
DNA packaging
chromatin (dis-)assembly
endomembrane organization
macroautophagy
telomer organization
protein-DNA complex subunit organization
vesicle organization
protein targeting
proteasomal protein catabolic process
chromatin remodelling
DNA conformation change
proteasome-mediated ubiquitin-dependent protein
vacuolar transport
lysosomal transport
pattern specification process
vesicle organization
response to vitamin
response to ER stress
autophagy
process utilizing autophagic mechanism
chromatin organization
negative regulation of developmental process
GeneRatio
Count
p.adjust
d)
endplasmatic reticulum to Golgi vesicle-mediated transport
DNA strand elongation involved in DNA replication
Ranked List Metric
Running Enrichment Score
Position in the Ranked List of Genes
e)
Neighbour gene expression
Z-score
ENSG00000266554
ENSG00000287723
ENSG00000186481
ENSG00000283294
ENSG00000286293
ENSG00000263618
ENSG00000266145
ENSG00000265787
ENSG00000266522
ENSG00000264301
ENSG00000264570
ENSG00000180777
ENSG00000265737
ENSG00000263821
Control_R1
Control_R2
sgRNA6_d3_R1
sgRNA6_d3_R2
sgRNA6_d5_R1
sgRNA6_d5_R2
sgRNA7_d3_R1
sgRNA7_d3_R2
sgRNA7_d5_R1
sgRNA7_d5_R2
f)
XLOC_030781 locus

Figure S5 continued

g) 501mel

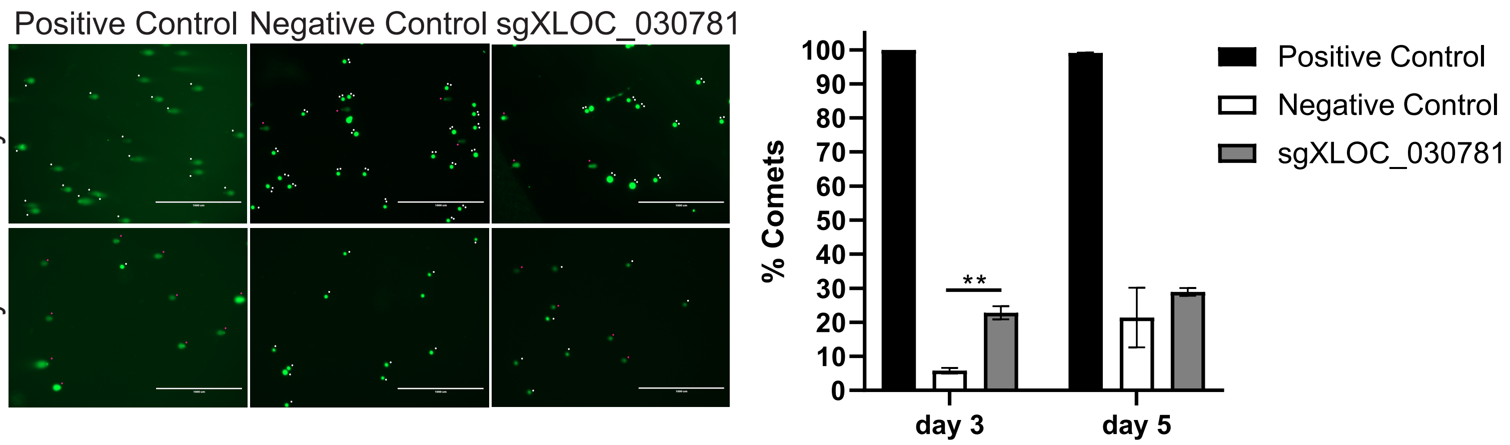

h) WM1361a

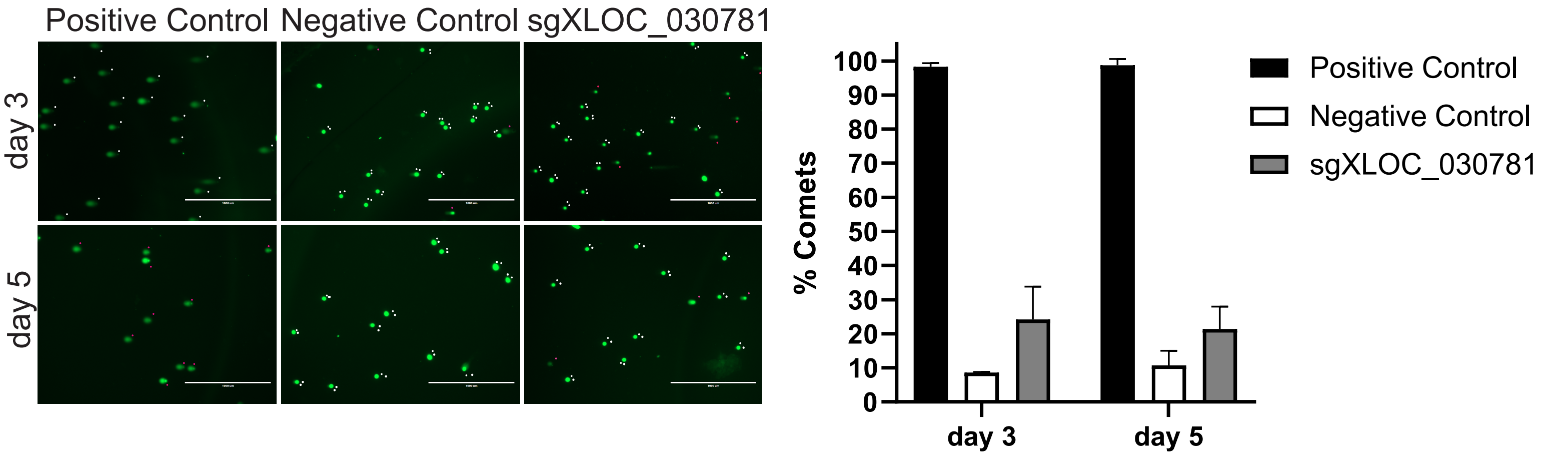

Figure S5:
XLOC_030781 CRISPRi knockdown and RNA-sequencing transcriptomic expression analysis in 501mel-dCas9-KRAB at day 5 and additional controls. a) Volcano plot of significantly differentially expressed genes (red) upon sgXLOC_030781 knockdown at day 5, p-value cut-off 0.05, log2 fc= 1. b) Volcano plot expression comparison of sgXLOC_030781 knockdown of day 3 vs. day 5. c) Gene ontology enrichment analysis of up- and down regulated gene sets upon sgXLOC_030781 knockdown at day 5. d) GSEA enrichment analysis of each top example of up-regulated (ER to Golgi vesicle transport, left) and down-regulated genes (DNA-strand elongation involved in DNA replication, right). e) Heatmap shown no significant neighbor gene expression correlation upon sgXLOC_030781 in 501mel-dCas9-KRAB at both time points day 3 and 5. f) IGV browser snapshot of ChIP-Seq analysis for H3K4me3 and H3K27ac and input control of the XLOC_030781 locus for representative melanocytes, BM, LN and WM1361a. Red boxes indicate identified peaks near the respective TSS. g) COMET assay for involvement of XLOC_030781 knockdown in DNA double strand break at day 3 and 5 post-infection in 501mel-dCas9-KRAB and h) WM1361a-dCas9-KRAB. Right panel shows COMET quantification of sgXLOC_030781 knockdown
