# Supplementary material for: Uncovering Novel lncRNAs Linked to Melanoma Growth and Migration with CRISPR Inhibition Screening
Source: Cancer Res Commun. 2025 Jul 9;5(7):1102–18. doi: 10.1158/2767-9764.CRC-24-0416 (PMC12238846; doi:10.1158/2767-9764.CRC-24-0416)
Supplement: Figure S6 — TCGA metaanalysis of lncRNA XLOC_030781 [file crc-24-0416_figure_s6_suppsf6.pdf]

Figure S6

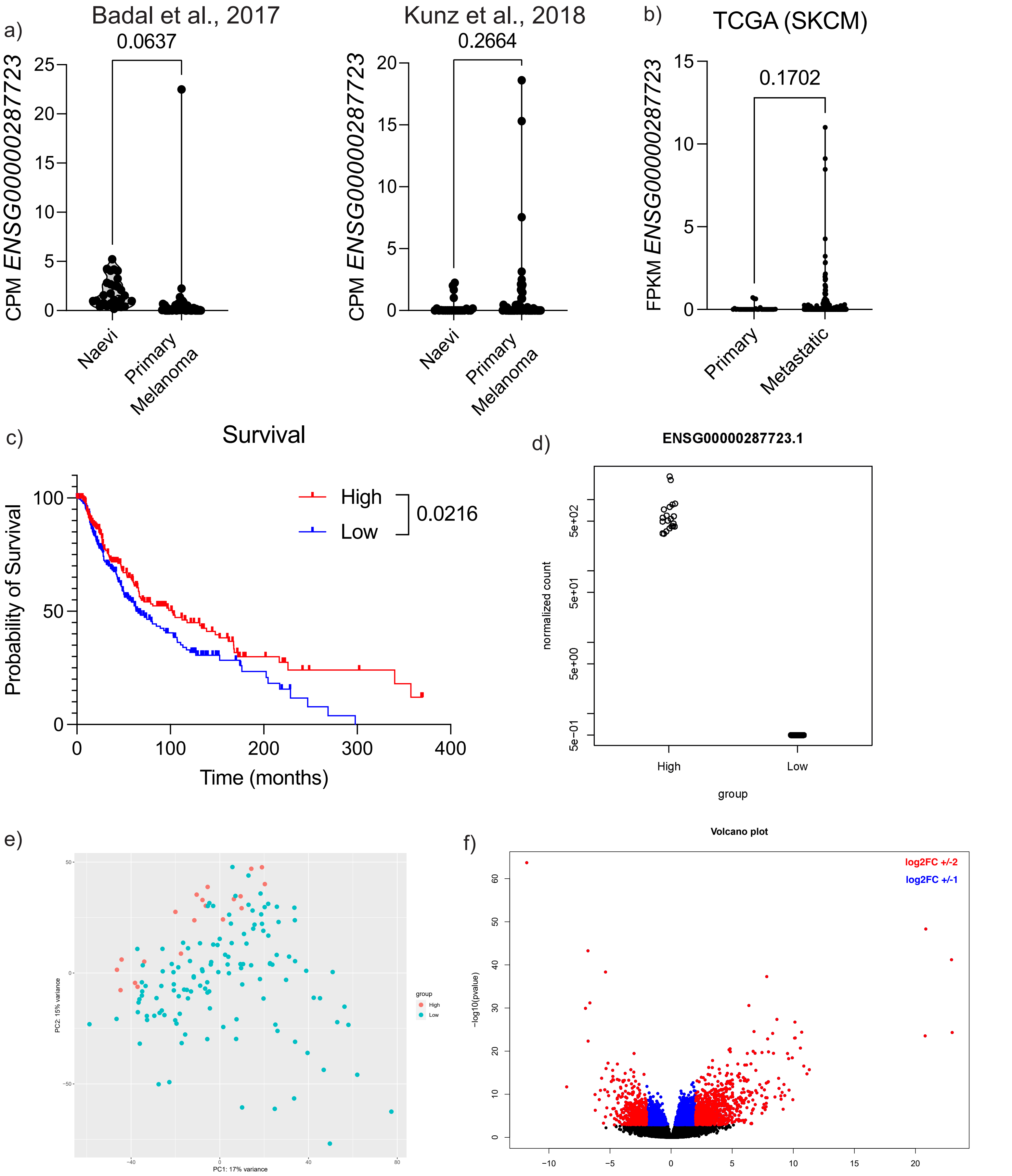

Figure S6:
a) XLOC_030781 expression analysis in RNA-Sequencing data analysis from Badal et al., 2017 naevi (n=27) vs. primary melanoma (n=51) (left), Kunz et al., naevi (n=23) primary melanoma (n=57)(right), unpaired t-test and b) TCGA of primary melanoma (n=76) vs. metastatic melanoma (n=367) c) Kaplan-Meier patient survival in XLOC_030781 high vs. low expression samples (Log-rank Mantel-Cox test). XLOC_030781/ENSG0000287723 lncRNA expression metanalysis in clinical melanoma TCGA samples by DESeq2. d) Stratification of 20 melanoma with very high vs 125 patients with absent XLOC_030781/ENSG0000287723 expression. e) PCA analysis of high vs. patients with absent XLOC_030781/ENSG0000287723 expression. f) Volcano plot of differentially expressed genes in high vs. patients with absent XLOC_030781/ENSG0000287723 expression.
